# Caregiver Burden among Patients with Influenza or Influenza-like Illness (ILI): A Systematic Literature Review

**DOI:** 10.3390/healthcare12161591

**Published:** 2024-08-09

**Authors:** Shailja Vaghela, Verna L. Welch, Anup Sinh, Manuela Di Fusco

**Affiliations:** 1 HealthEcon Consulting, Inc., Ancaster, ON L9G 4L2, Canada; shailja@healthecons.com (S.V.);; 2 Pfizer, Inc., New York, NY 10001, USA; verna.welch@pfizer.com

**Keywords:** caregiver burden, health-related quality of life (HRQoL), influenza, influenza-like-illness, productivity loss, systematic literature review, work productivity

## Abstract

Influenza and influenza-like illness (ILI) pose significant clinical and economic burdens globally each year. This systematic literature review examined quantitative studies evaluating the impact of patients’ influenza/ILI on their caregivers’ well-being, focusing on health-related quality of life (HRQoL), work productivity, and activity impairment. A comprehensive search across six databases, including the Cochrane Database of Systematic Reviews, Embase, MEDLINE via PubMed, Ovid, PsycNet, and Web of Science, yielded 18,689 records, of which 13,156 abstracts were screened, and 662 full-text articles were reviewed from January 2007 to April 2024. Thirty-six studies [HRQoL: 2; productivity: 33; both: 1] covering 22 countries were included. Caregivers of 47,758 influenza or ILI patients across 123 study cohorts were assessed in the review. The mean workday loss among caregivers ranged from 0.5 to 10.7 days per episode, influenced by patients’ influenza status (positive or negative), disease severity (mild or moderate-to-severe), age, viral type (influenza A or B), and vaccination/treatment usage. The HRQoL of caregivers, including their physical and emotional well-being, was affected by a patient’s influenza or ILI, where the severity and duration of a patient’s illness were associated with worse HRQoL. This review shows that the consequences of influenza or ILI significantly affect not only patients but also their caregivers.

## 1. Introduction

Influenza has a longstanding history of causing substantial annual morbidity and mortality due to the virus’s variability and widespread distribution among humans, birds, swine, and other mammals [1]. Annually, there are an estimated one billion cases of influenza in the general population globally, of which three to five million cases are severe [2]. The impact of influenza or influenza-like illness (ILI) extends beyond the immediate health consequences, imposing prominent clinical and economic burdens on patients, their families, and society at large [3,4]. 

Influenza or ILI causes significant economic impact on society, encompassing both direct healthcare costs (e.g., hospitalizations, outpatient visits) and indirect costs (e.g., productivity loss). Health-related productivity loss has been primarily driven by short-term and long-term absenteeism for patients and their caregivers, depending on the duration and severity of illness, as well as by the effects of outbreak-related restrictions, presenteeism, and the inability to engage in unpaid work due to illness [5,6]. Moreover, these illnesses affect health-related quality of life (HRQoL), influencing not only patients but also considerably affecting the well-being of their informal caregivers, defined as individuals offering care and assistance without financial compensation to their family members and friends with influenza or ILI [7,8].

While the impact of influenza or ILI on patients has been well-documented, there is a notable gap in systematically reviewing their effects on caregiver well-being, particularly concerning HRQoL and the productivity loss experienced by caregivers due to a patient’s illness [6,7]. Currently, there is no systematic literature review (SLR) available to assess HRQoL among informal caregivers due to a patient’s illness, while only one review by Zumofen et al., 2023 assessed the burden of influenza or ILI on the work productivity of patients and caregivers, without exclusively focusing on the burden among informal caregivers due to their family members or friends’ influenza or ILI [6]. Estimating the impact on caregivers’ work productivity in terms of reduced work hours, missed workdays, or even job loss due to the demands of caregiving can help in characterizing the broader economic burden of influenza or ILI beyond the affected patients, identifying unmet needs and potentially quantifying the health economic value of health interventions. Hence, to fully comprehend the overall societal burden of illness and to assess the societal benefits of interventions and vaccination, it is essential not only to study how they affect patients but also to thoroughly examine their impact on caregivers. 

This literature review aimed to systematically collect and synthesize available evidence on the burden experienced by informal caregivers of patients with influenza or ILI. Specifically, the study objective was to synthesize studies that assess the impact of influenza or ILI on caregivers’ well-being, focusing on their HRQoL, work productivity, and activity impairment due to the patient’s illness.

## 2. Materials and Methods

This SLR was designed and conducted following standard systematic review guidance, such as the CRD’s guidance for undertaking reviews in healthcare and the JBI manual for evidence synthesis [9,10]. A protocol outlining the objectives, inclusion and exclusion criteria, search strategy, and methods of analysis was developed a priori. The review is reported in accordance with the Preferred Reporting Items for Systematic Reviews and Meta-Analyses (PRISMA) 2020 Statement, which provides comprehensive guidelines for reporting SLRs [11]. 

### 2.1. Eligibility Criteria and Definitions

The Population, Intervention, Comparator, Outcome, Study design (PICOS) and other eligibility criteria are described in Supplementary Materials Table S1. The targeted population was informal caregivers of patients with laboratory-confirmed influenza, physician-reported influenza or ILI, and self/caregiver-reported influenza or ILI. Classification criteria for influenza or ILI were used according to the terminology utilized in the studies, either with or without a clearly delineated set of symptoms specified by the study authors. For instance, according to the World Health Organization (WHO), ILI is characterized by an acute respiratory infection with a measured fever of ≥38 °C and cough, with onset within the last 10 days [12]. However, study investigators from various geographical regions may adopt different definitions or criteria to define influenza or ILI. Nonetheless, we categorized those populations as deemed appropriate for the review. Similarly, we relied on the study authors’ reported definitions or terminology for the episode of infection or severity of the disease without further investigation.

The study population exclusively targeted informal caregivers, defined as individuals who offered care and assistance to family members and friends with influenza or ILI without financial compensation [8]. Professional caregivers in healthcare settings, such as long-term care facilities and nursing homes, were excluded. Moreover, caregivers face an increased risk of contracting influenza or ILI from their ill family members (referred to as secondary infection), which can impact both their work productivity and QoL [13]. It was defined a priori that this review would exclusively examine the burden among informal caregivers because of a patient’s illness.

The outcomes of interest were HRQoL, work productivity, and activity impairment among informal caregivers due to a patient’s influenza or ILI. The HRQoL outcomes included health disutilities (representing a decrement in the valued quality of life) related to overall HRQoL or impacts on different domains such as physical functioning, psychological domain, and social functioning among caregivers, assessed by various patient-reported outcome (PRO) instruments or quantitative surveys [13,14]. Qualitative studies focusing solely on opinions, concepts, or themes without providing relevant quantitative data were excluded. 

Activity impairment and work productivity outcomes included absenteeism, presenteeism, or related measures such as the amount of time devoted to caregiving or time lost for leisure activities. Productivity outcomes assessed by primary studies (e.g., real-world evidence (RWE) claim databases, questionnaires, or surveys) were deemed eligible for inclusion, whereas modeled outcomes derived from the literature were excluded.

Absenteeism was characterized as the loss of productivity resulting from the absence from work due to caring for an ill household member, whereas presenteeism referred to diminished productivity experienced while attending work despite a household member’s illness [15,16].

The review included various types of empirical research study designs, excluding non-research publications or modeled studies. Prior SLRs were screened to identify relevant studies; however, data extraction was not directly performed from these prior reviews. The literature search was restricted to studies published in English from 1 January 2007 to 30 April 2024, without any geographical limitations, to capture relevant caregiver-related studies and to cover more than 25 influenza seasons.

### 2.2. Source of Information

The systematic literature search covered databases of Cochrane Database of Systematic Reviews, Embase, MEDLINE via PubMed, Ovid, APA PsycNet (including PsycInfo and PsycExtra), and Web of Science. Additionally, a hand search was conducted using MedRxiv and Google Scholar. The search strategy employed for databases is detailed in Supplementary Materials Tables S2–S4.

### 2.3. Screening, Data Extraction, and Synthesis 

Two independent reviewers conducted a double-blind screening of titles and abstracts to ensure internal quality control. Disagreements regarding inclusion or exclusion were resolved through discussions between the reviewers. Articles identified as potentially relevant following the initial title and abstract screening underwent a thorough full-text review. Subsequently, inclusion decisions for a final list of studies were independently made by two researchers, followed by a consensus discussion to reconcile any disparities.

Data extraction was performed using the predetermined Microsoft 365 Excel spreadsheet by one reviewer, followed by a quality check of extracted data conducted by a second reviewer. Information was extracted at the study cohort level as reported by the authors. Supplementary Materials Tables S5–S9 represent the key data fields extracted from the selected studies. 

Various types of study designs were utilized in the identified studies, including retrospective and prospective observational studies, cross-sectional studies, and randomized controlled trials (RCTs). Consequently, the research findings were synthesized narratively, allowing for a detailed and qualitative interpretation of the data. In addition, the identified studies reported caregiver-specific outcomes for overall participants and/or stratified by subgroups, including influenza status, severity of the disease, vaccination status among patients with influenza or ILI. Hence, we considered each study with its overall sample or relevant sub-cohorts as the study cohorts to describe the caregivers’ outcomes. Descriptive statistics of the collected data were also reported to provide a quantitative overview of the study outcomes. For the HRQoL outcomes among caregivers, quantitative findings in terms of total score and domain-specific scores were described as reported by the study authors. For the work productivity outcomes in the selected studies, the total cohort (N) size representing the number of patients examined in the respective study sub-cohort, the proportion of caregivers reporting absenteeism, and the number of missed workdays [mean (SD) and/or median (IQR)] were presented in tables and figures. The total cohort N of patients also served as a proxy if the number of caregivers was missing in that study sub-cohort. Due to heterogeneity among the studies and limited caregiver-specific details, no feasibility assessment of conducting a meta-analysis nor a risk of bias assessment were performed.

## 3. Results

### 3.1. Study Selection

**Figure 1:**
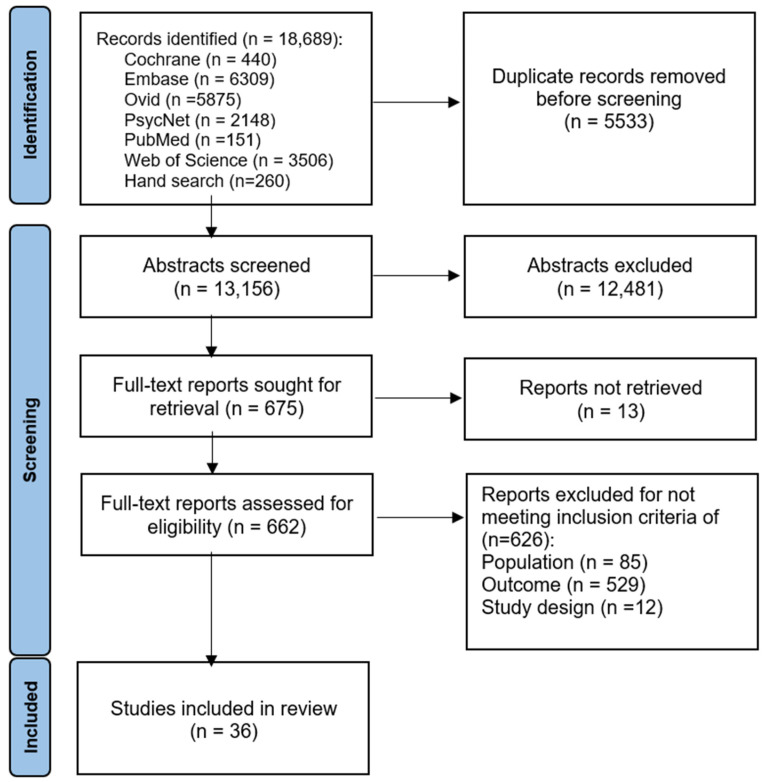
PRISMA flowchart of identification and selection of studies in the systematic review. PRISMA, Preferred Reporting Items for Systematic Reviews and Meta-Analyses. Note: PRISMA template was adapted from [11].

A total of 18,689 records were identified through database searches from 1 January 2007 to 30 April 2024, from which 13,156 (70.4%) abstracts and titles were screened after removing 5533 duplicate abstracts. Subsequently, 675 (3.6%) full-text articles were reviewed, of which 36 studies, comprising 2 for HRQoL, 33 for productivity outcomes, and 1 for both outcomes, met the inclusion criteria and were included in the systematic review (Figure 1).

### 3.2. Characteristics of the Selected Studies in the Review

**Figure 2:**
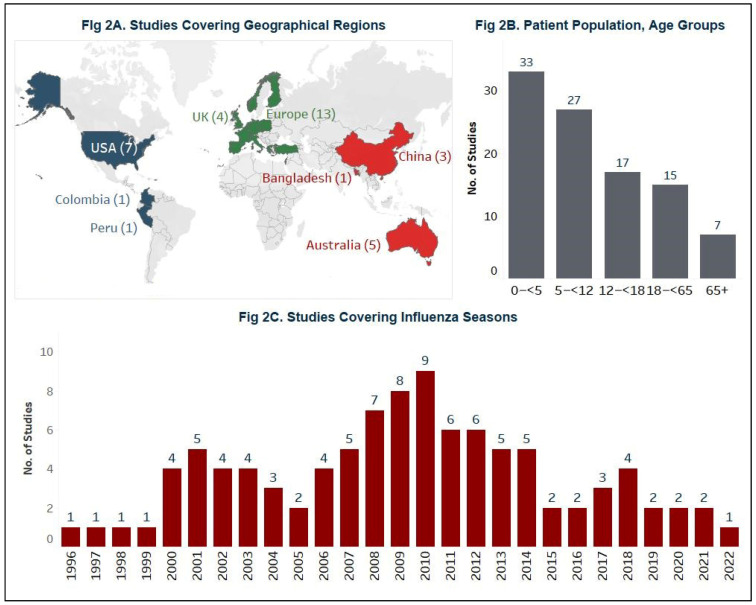
Study characteristics of the selected studies in the review. (**A**) represents the number of studies covering geographic regions. (**B**) represents the number of studies assessing caregivers of patients with influenza or ILI (by age group). (**C**) represents the number of studies covering influenza seasons as reported in the selected studies.

Thirty-six studies from various regions, including Europe (n = 13), the United States (US, n = 7), and China (n = 5) were included in the review (Figure 2A). A wide range of study designs were employed to assess the caregiver burden, including retrospective or prospective observational studies, cross-sectional surveys, and RCTs. Most of the studies focused on caregivers of pediatric patients, especially young children aged <5 years (n = 33) and those aged 5 to 12 years (n = 27), whereas only seven studies addressed caregivers of elderly patients aged 65+ years (Figure 2B). The reviewed studies span influenza seasons from 1996 to 2022. Notably, the influenza season of 2010 was covered most extensively, represented in nine studies, followed by the seasons of 2009 (n = 8) and 2008 (n = 7) (Figure 2C). Detailed study characteristics, including study methodology, setting, patient population, study population, outcomes, and instruments/questionnaire used to collect data, are described in Supplementary Materials Table S5–S8. 

### 3.3. Impact on Health-Related Quality of Life (HRQoL) of Caregivers Due to Patients’ Influenza or ILI

Three studies, including two from Australia and one from the US, examined HRQoL among 717 caregivers of a total of 717 patients across six study cohorts (Table 1, Tables S5 and S6). Two studies included 95% mothers and 5% fathers [17,18], while the study cohort in Overmann et al., 2023 comprised 93.9% mothers, 5% fathers, and 1.1% grandmothers [7]. All three studies reported that more than 60% of caregivers were working during the study period. Table 1 describes the total scores and domain-specific scores of HRQoL among caregivers assessed by the reported instruments, while Supplementary Materials Tables S5 and S6 describe study details including caregiver-specific details and instruments used in the identified studies. 

#### 3.3.1. Australia

Chow et al., 2013a developed and validated a disease-specific measure called the quality of life of caregivers of children with influenza-like illness (Care-ILI-QoL) through an RCT conducted during the influenza season of 2011 in Australia. This assessment tool comprised 16 items covering four domains: daily activities, perceived support, social life, and emotions. Scores derived from the Care-ILI-QoL were computed for each domain and in total, with a score of 7 representing the highest possible quality of life and 1 indicating the lowest [17]. 

Among the caregivers of 55 children aged 6 to 48 months with ILI, the mean (SD) age was 33.7 (3.9) years, and the annual household income was USD 123,972 (52,759). The total QoL score was 3.87 (0.93), with the social life domain recording the lowest mean score of 3.24 (0.84). Significant score differences were observed among caregivers with different perceived severity levels of their child’s ILI: F(2, 71) = 5.8, *p* = 0.007. Caregivers who perceived their child as ‘very/extremely sick’ [N = 17, mean (SD) = 3.36 (1.11)] reported significantly lower total QoL scores compared to those perceiving their child as ‘mildly sick’ [N = 23, mean (SD) = 4.28 (0.58); *p* = 0.005]. Additionally, significant score differences were noted among caregivers who dedicated varying durations to caring for their child: F(2, 71) = 3.3, *p* = 0.044. C1.04] who devoted 10 h or more [N = 18, mean (SD) = 3.48 (1.04)] reported significantly lower total QoL scores than those who had not devoted any time [N = 37, mean (SD) = 4.11 (0.91)], although not significantly lower than those who spent 1–9 h [n = 19, mean (SD) = 3.75 (0.71)]. Furthermore, significant score differences were observed among caregivers with different numbers of general practitioner (GP) visits: F(2, 67) = 12.49, *p* < 0.001. Caregivers whose child had two or more GP visits reported lower total QoL scores [N = 19, mean = 3.15 (0.85)] compared to those with no visits [N = 28, mean (SD) = 4.32 (0.15); *p* < 0.001] and one visit [N = 23, mean (SD) = 3.81 (0.73); *p* = 0.023] [17].

Chow et al., 2013b conducted a prospective cohort study before the influenza season of 2010 (26 Mar–30 Jul, baseline) and during the influenza season (30 Jul–15 Nov, post-ILI) to compare HRQoL among caregivers of children aged 6 to 36 months with ILI (ILI group) to those of age-matched children without ILI (non-ILI group). The study utilized the Caregiver’s Quality of Life as related to Ear, Nose, and Throat (PAR-ENT-QoL), a 15-item questionnaire covering emotional and daily disturbance domains, administered at baseline and post-ILI. Additionally, the Short Form Health Survey version 2 (SF-12v2) was administered post-ILI. PAR-ENT-QoL items were rated using a five-point Likert scale, with scores reversed for consistency with the SF-12v2 acute Form (i.e., scoring from 0 to 100, where a higher score represents better QoL) [18]. 

Out of the 381 children enrolled across 90 childcare centers in Australia, 105 developed ILI. Among caregivers of the ILI group, with a mean (SD) age of 36 (4.1) years [vs. 36 (4.3) years in the non-ILI group], 79% reported having a university education or higher [vs. 67% in the non-ILI group]. Further, 75% of both parents were working and 77% of caregivers reported a weekly household income of more than AUD 2000 compared to 72% and 77% of caregivers in the non-ILI group, respectively. Post-ILI follow-up interviews showed a significant decline in PAR-ENT-QoL scores for the ILI group compared to baseline (60.99 vs. 79.77, *p* < 0.001), as well as to the non-ILI group during follow-up (60.99 vs. 84.05, *p* < 0.001). Furthermore, SF-12v2 scores indicated notably lower outcomes for the ILI group compared to the non-ILI group, both in the physical component summary (50.66 vs. 53.16, *p* = 0.011) and the mental component summary (45.67 vs. 53.66, *p* < 0.001). Moreover, the impact on caregivers’ QoL remained consistent across each ILI episode. Par-ENT-QoL scores did not significantly differ between the first and second episodes [N = 89 vs. 16; total QoL score: 58.61 vs. 55.53, *p* = 0.60; emotional domain: 59.86 vs. 53.37, *p* = 0.26; daily disturbance: 58.33 vs. 57.74, *p* = 0.93]. Additionally, multi-regression analysis revealed that two factors, ‘total time spent caring for the child during ILI (β = −0.24, *p* = 0.03)’ and ‘severity of ILI (β = 0.20, *p* = 0.04)’, were significantly associated with PAR-ENT-QoL total scores. Specifically, with each additional 10.4 h devoted to caring for the child during ILI, the total QoL score decreased by 0.24 SD units [18].

#### 3.3.2. United States

Overmann et al., 2023 [7] assessed the HRQoL among US caregivers of children aged 6 to 48 months with ILI presenting to either an urgent care (UC) or emergency department (ED) during February 2020 to May 2021. The Care-QoL-ILI, developed by Chow et al., 2013a [17], was administered to 281 participants. The majority of caregivers (91%) were in the age group of 20–40 years. Additionally, 36% of caregivers reported having a college or post-graduate degree, 64% reported being employed, and 33% reporting an annual household income of less than USD 15,000. Caregivers reported a decline in QoL during their child’s ILI, with a mean (SD) overall QoL score of 3.90 (1.52). Notably, the lowest QoL was reported in the emotion domain [mean (SD): 2.80 (1.58)], indicating significant concern among caregivers regarding their child’s illness and its potential transmission to the family. Furthermore, caregivers who perceived a worse illness severity (very sick vs. mildly sick) had lower scores in the emotions domain (2.61 vs. 6.00, *p*  =  0.0269) and daily life disturbance (3.87 vs. 4.83, *p* = 0.0065), while illness duration was associated with poorer overall QoL scores, worsening by 0.128 for each additional day of illness [7].

### 3.4. Impact on Work Productivity of Caregivers Due to Patients’ Influenza or ILI

Thirty-four studies assessing the impact on work productivity of caregivers due to patients’ influenza or ILI were identified, primarily focusing on absenteeism among caregivers (n = 34), while only three studies evaluated presenteeism and its impact on caregivers’ leisure time. The collective studies represented a total of 47,758 patients with influenza or ILI across 117 study cohorts, covering 22 countries from diverse geographic regions. No studies from any African countries were identified in this review. All the identified studies, except Overmann et al., 2023 [7], included caregiver-related outcomes alongside the outcomes for the infected patients without exclusively focusing on caregiver burden. Specifically, only 16 studies out of the 34 studies reported some caregiver-specific details, while 18 studies did not provide any caregiver-specific demographics or other information. Most of the studies measured work productivity outcomes among caregivers, reporting the percentage of caregivers missing work and the number of workdays loss [mean (SD) or median (IQR)], using different measurement methods such as structured questionnaires (n = 14) [7,19,20,21,22,23,24,25,26,27,28,29,30,31], surveys (n = 6) [32,33,34,35,36,37], or interviews (n = 8) [38,39,40,41,42,43,44,45]. However, none of the studies reported using standard validated tools like the Work Productivity and Activity Impairment (WPAI) [46]. Detailed study characteristics, reported caregiver-specific details, and study results are described in Supplementary Materials Tables S7–S9. 

#### 3.4.1. America

**Figure 3:**
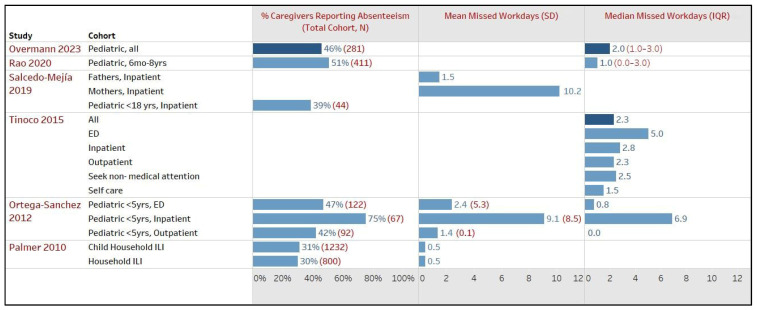
Impact on work productivity of caregivers due to patients’ influenza or influenza-like illness (ILI), America. ED, emergency department; ILI, influenza-like-illness; IQR, inter quartile range; SD, standard deviation. Note—The total cohort (N) represents the number of patients with influenza or ILI examined in the respective study sub-cohort. For example, Ortega-Sanchez 2012 included a total of 281 children with influenza, with 122 in the sub-cohort of pediatric <5 years. The total cohort (N) also serves as a proxy if the number of caregivers was not explicitly reported in that study sub-cohort [7,25,29,35,44,45].

Nine studies, including seven from the US and one each from Peru and Colombia, examined work productivity outcomes among caregivers affected by their family members’ influenza or ILI. Across these nine studies, which collectively involved 17,220 patients across 19 study cohorts, work absenteeism was consistently reported, with two studies also providing data on presenteeism and unpaid activities resulting from the family members’ illness. Figure 3 depicts six American studies that reported the proportions of caregivers who missed work and the mean or median days of missed work [7,25,29,35,44,45]. 

In the US, the rate of caregiver absenteeism varied widely depending on the severity of a patient’s illness, ranging from 24% to 75%, with a substantial 75% absenteeism rate reported for caregivers of hospitalized patients. The duration of missed workdays also showed considerable variability, ranging from a mean (SD) of 0.5 (NR) to as high as 9.1 (8.5) days for caregivers of hospitalized patients [7,35,38,43,44,45]. Li et al., 2007 conducted a retrospective cohort study of 12,850 healthy US households with children (5–17 years). In these households, more than 80% were medically insured, 98% were non-retired, and over 25% were unemployed. The ILI households lost 1.12 more workdays (95% CI: 0.20–2.04, *p* < 0.05) vs. non-ILI households [34]. 

Similarly, in Colombia, the average number of days caring for hospitalized children with influenza-confirmed severe acute respiratory infection was 10.2 days (95% CI, 5.4–14.9) among mothers and 1.5 days (95% CI, 0.6–2.4) among fathers [25]. Tinoco et al., 2015, in Peru, assessed 1321 influenza cases and found that workplace-related absenteeism among caregivers for total cases had a median (IQR) of 2.3 (2.5) days, ranging from 1.5 (1.5) days for patients who did not seek any medical care to 2.8 (NR) days for hospitalized patients and 5.0 (4.6) days for those treated in emergency departments (no *p*-values reported). Additionally, Tinoco et al. reported a median (IQR) of 1 (1) unpaid activity days overall, including 0.8 (0.75) days for patients who did not seek medical care and 3.8 (4.8) days for caregivers of hospitalized patients [29].

Palmer et al., 2010 in the US surveyed employees [mean (range) age: 41.7 (21–64)], comprising 86% white, 31% female, and 41% with a college degree, to assess absenteeism and presenteeism when their household members had ILI. The reported mean (SD) hours of ILI-related presenteeism among individuals when household members had ILI and individuals with children in the household affected by ILI were at 1.3 (3.5) and 1.4 (3.7) hours, respectively, while both groups reported an average of 0.5 missed workday(s) [35]. 

#### 3.4.2. Asia–Pacific

**Figure 4:**
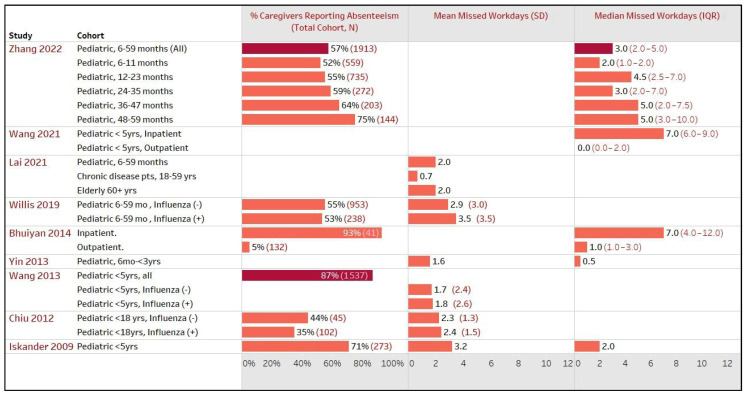
Impact on work productivity of caregivers due to patients’ influenza or influenza-like illness (ILI), Asia–Pacific. Note—The total cohort (N) represents the number of patients with influenza or ILI examined in the respective study sub-cohort. For example, Willis 2019 included a total of 1191 pediatrics aged 6–59 months, with 953 and 238 in the sub-cohorts of influenza-positive and -negative, respectively. The total cohort (N) also serves as a proxy if the number of caregivers was not explicitly reported in that study sub-cohort [19,20,22,30,31,37,42,47,48].

Nine studies from the Asia–Pacific (APAC) region, comprising five from China [20,22,30,37,47], three from Australia [31,42,48], and one from Bangladesh [19], reported work productivity impairment among caregivers of 9640 patients (study cohorts n = 22) across the region. Most of the studies (n = 6) did not provide caregiver-specific details, while two studies only reported household incomes. The proportion of caregivers reporting absenteeism due to a family member’s illness varied widely, ranging from 35 to 87% in China [20,30,37], 53 to 71% in Australia [31,42], to 5 to 100% in Bangladesh [19]. As shown in Figure 4, the mean (SD) of missed workdays ranged from 0.7 (NR) to 3.5 (3.5) among the six APAC studies, while three studies reported a median (IQR) of up to 7 (4.0–12.0) days. 

The number of missed workdays also varied significantly depending on the patient cohorts, encompassing inpatients or outpatients, influenza-positive or -negative cases, influenza A or B, and different age groups. Caregivers of inpatients with influenza or ILI reported the highest number of missed workdays compared to those of outpatients, with a median (IQR) of 7.0 (4.0–12.0) vs. 1.0 (1.0–3.0) in Bangladesh [19] and 7.0 (6.0–9.0) vs. 0.0 (0.0–2.0) in China [47]; however, these studies did not report *p*-values. A study by Lai et al., 2021 in China reported the mean (95% confidence interval) missed workdays for caregivers of children aged 6–59 months, chronic disease patients aged 18–59 years, and the elderly aged 60+ years at 2 (1.9–2.1), 0.7 (0.4–1.0), and 2 (1.5, 2.6), respectively [22].

Only one Australian study by Yin et al., 2013, where 73% of both parents were working and 75% of had a weekly household income >USD 20,000, reported ILI-related time away from recreation for caregivers, with a mean of 3.1 h (median 0 h) [48].

#### 3.4.3. Europe and the United Kingdom 

Twelve studies conducted across European countries [21,23,24,26,27,32,39,40,41,49,50,51], three from the United Kingdom (UK) [28,33,36], and one study by Ambrose and Antonova 2013 covering multiple European countries, the UK, and Israel, were included in the review [52]. While all studies reported workday losses among caregivers, no European study assessed presenteeism or the impact on leisure and unpaid activities. However, Aykac et al., 2017 in Turkey reported the proportions of sleep disturbances among caregivers due to children’s influenza or ILI [49].

##### United Kingdom

**Figure 5:**
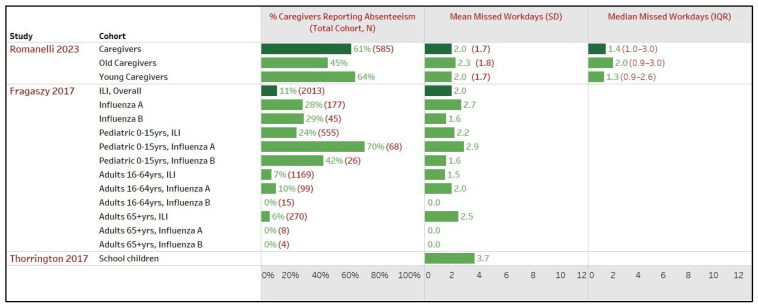
Impact on work productivity of caregivers due to patients’ influenza or influenza-like illness (ILI), United Kingdom. Note—Romanelli 2023 defined old caregivers as working adults aged 50–64 years and younger caregivers as working adults aged 18–49 years who cared for patients with influenza or ILI during the survey. The total cohort (N) represents the number of patients with influenza or ILI examined in the respective study sub-cohort. For example, Fragaszy 2017 included a total of 2103 patients with ILI, with 177 for influenza A. The total cohort (N) also serves as a proxy if the number of caregivers was not explicitly reported in that study sub-cohort [28,33,36].

In the UK, three studies covered a total of 2632 patients with influenza or ILI across 16 study cohorts (Figure 5). Romani et al., 2023 conducted a geographically representative survey of 1000 individuals in the UK. Among the 585 survey respondents who reported caring for a dependent with influenza while employed, 61% reported taking an average of 2.0 days off work [SE ± 1.7, median (IQR): 1.4 (1–3) days]. Older working adults were less likely to take time off from work but for a longer mean (SD) duration than younger adults [50–64 yrs: 44.9%, 2.3 (1.8) days; 18–49 yrs: 63.5%, 2.0 (1.7) days, no *p*-values reported] [36]. Fragaszy et al., 2017, covering six influenza seasons from 2006 to 2011, assessed the impacts on caregivers for 2013 individuals with ILI of different age groups and 3161 individuals tested for influenza type A or B. Overall, 11% of caregivers reported taking time off, averaging 2.0 days (min–max: 1–7), which varied widely depending on the age of the patient [0–15 yrs: 24%, 2.2 (1–7) days; 16–64 yrs: 7%, 1.5 (1–5) days; 65+ yrs: 6%, 2.5 (1.5) days] and type of influenza [influenza A: 28%, 2.7 (1–6) days; influenza B: 29%, 1.6 (1–2) days]. However, *p*-values were not reported for any group [33]. Conversely, Thorrington et al., 2017 reported a higher mean (95% CI) of 3.7 (95% CI: 2.7–4.8) workdays lost among caregivers of 34 children with ILI symptoms compared to the findings of the other two studies [28]. However, Romani et al., 2023 and Fragaszy et al., 2017 did not provide caregiver-specific demographics, while Thorrington et al., 2017 did not report the percentage of caregivers reporting absenteeism. Hence, a comparison among the study cohorts of all three UK studies was not feasible [28,33,36].

##### Europe

A total of 18,266 patients and their caregivers were investigated in European studies (including 5656 for Ambrose and Antonova 2013), covering 14 countries. Of the 13 European studies, only 4 studies provided some caregiver-related information, including 2 from the Netherlands and 1 each from Germany and Turkey. The proportion of caregivers affected by a patient’s illness, as well as the impact on their work productivity, varied widely depending on factors such as the influenza status (positive or negative), severity of the condition, age group, viral type, and vaccination status among patients with influenza or ILI. 

Approximately 2% of parents, up to 100% of households, reported workday losses, ranging from a mean (SD) of 1.2 (1.4) days among fathers of children with influenza A/H1N1 in Italy to 10.7 (14.1) days among parents of hospitalized patients with influenza A in Spain. Most of the European studies focused on pediatric patients, with only two studies by Galante et al., 2012 and Silva et al., 2014 targeting patients of any age group, albeit covering approximately 30% and 70% pediatric patients, respectively.

##### Impact of Disease Severity of Patient’s Influenza or ILI on Work Productivity of Caregivers, Europe 

**Figure 6:**
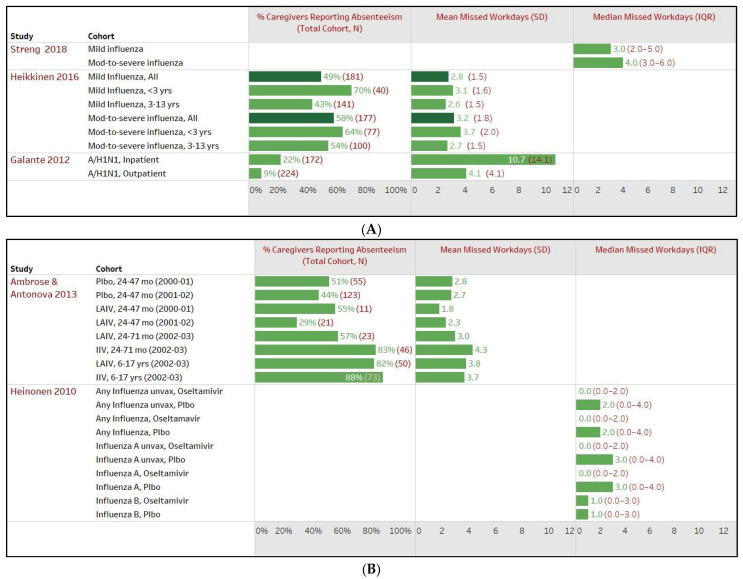

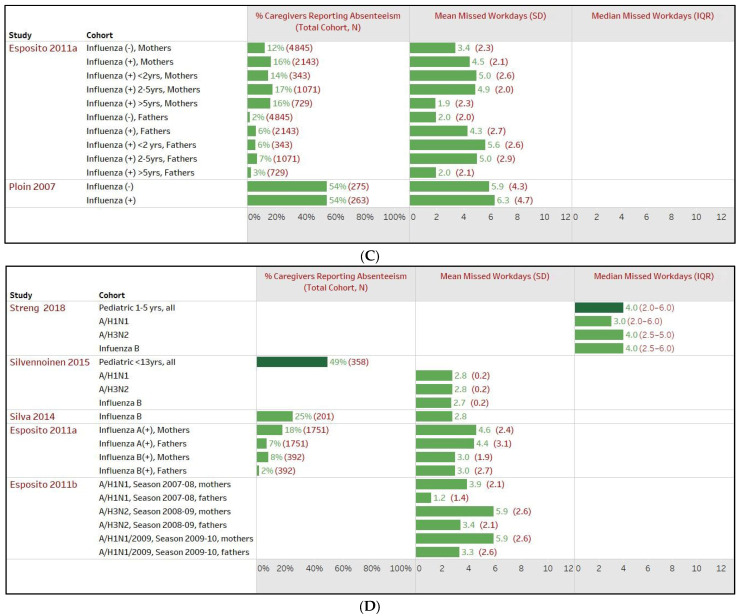
Impact of patients’ influenza or influenza-like illness (ILI) on work productivity of caregivers in Europe. (**A**) Impact of patient’s disease severity [21,27,41]. (**B**) Impact of patient’s vaccination status/treatment usage [50,52]. IIV, inactivated influenza vaccine; LAIV, live attenuated influenza vaccine; Plbo, placebo. (**C**) Impact of patients’ influenza status (positive or negative) [24,39]. (**D**) Impact of patients’ influenza viral type (influenza A or B) [26,27,39,40,51]. Note—The total cohort (N) represents the number of patients with influenza or ILI examined in the respective study sub-cohort. For example, Galante 2012 in (**A**) included a total of 396 patients with ILI, with 172 and 224 in the sub-cohorts of inpatients and outpatients, respectively. The total cohort (N) also serves as a proxy if the number of caregivers was not explicitly reported in that study sub-cohort.

Three studies, comprising 794 patients across 10 study cohorts, examined impacts on caregivers’ productivity based on the severity of their condition, either as having mild or moderate-to-severe influenza or ILI, or as inpatients or outpatients (Figure 6A). The findings indicate that patients with a severe condition had a more significant impact on caregivers, with 22–70% of caregivers reporting an average loss of workdays ranging from 2.7 (1.5) to the highest reported in any patient group at 10.7 (14.1) days, compared to caregivers of mildly sick patients, where 9–70% reported 2.6 (1.5) to 4.1 (4.1) days of workdays lost [21,27,41]. Streng et al., 2018 reported median (IQR) absent days for mild influenza patients at 3 (2–5) days compared to patients with moderate-to-severe influenza [4 (3–6) days, *p* = 0.348] [27]. Heikkinen et al., 2016 reported *p*-values of 0.11, 0.17, and 0.86 for all children, <3 years and 3–13 years, respectively [21], whereas Galante et al., 2012 did not report *p*-values [41]. 

##### Impact of Vaccination Status or Treatment Usage among Patients with Influenza or ILI on the Work Productivity of Caregivers, Europe

Two studies examined the impact of vaccination with live attenuated influenza vaccine (LAIV) versus placebo or inactivated influenza vaccine (IIV), as well as early Oseltamivir treatment, on work productivity among caregivers (covering 6064 patients across 18 study cohorts) using data from RCTs (Figure 6B). 

Ambrose and Antonova 2013 analyzed data of vaccinated and unvaccinated children from three RCTs: Study 1 included children aged 6–35 months comparing LAIV vs. placebo (n = Yr 1: 490, 356; Yr 2: 570, 403), Study 2 involved children aged 24–71 months comparing LAIV vs. IIV (n = 790; 818), and Study 3 focused on children aged 6–17 years with asthma comparing LAIV vs. IIV (n = 1114; 1115). In Study 1, 55% of caregivers of children receiving LAIV reported an average of 1.8 missed workdays in year 1 and 29% reported 2.3 days in year 2, compared to the placebo cohort, wherein 51% of caregivers reported 2.8 days of loss in year 1 and 44% reported 2.7 days in year 2. Study 2 reported an average of 3.0 workdays lost among 57% of caregivers of children receiving LAIV compared to 4.3 days in 83% of caregivers of children receiving IIV. In Study 3, 82% of caregivers in the LAIV group experienced 3.8 days of work loss compared to 3.7 days in 88% of caregivers in the IIV group. However, no studies reported *p*-values for LAIV vs. placebo/IIV groups [52]. 

Heinonen et al., 2010 assessed 98 children with lab-confirmed influenza in a randomized trial, with 203 children receiving oseltamivir and 205 receiving a placebo. Oseltamivir treatment initiated within 24 h reduced parental work absenteeism by up to 3.0 days [for any influenza overall or among the unvaccinated: oseltamivir median (IQR): 0 (0–2) vs. placebo: 2 (0–4), *p* = 0.01 and *p* = 0.02, respectively; for influenza A overall or among the unvaccinated: oseltamivir: 0 (0–2) vs. placebo: 3 (0–4) days, *p* = 0.07 and 0.01, respectively; 1.0 (0–3) days for both groups in influenza B, *p* = 0.97] [50].

##### Impact of Patient’s Influenza Status (Positive or Negative) on Work Productivity of Caregivers, Europe

Three studies reported work absenteeism among caregivers covering 7526 patients across 12 study cohorts with positive or negative influenza status (Figure 6C depicts two of the three studies). Esposito et al., 2011a assessed cohorts of 2143 and 4845 Italian patients with influenza-positive or -negative status, respectively. Among influenza-positive patients, 16.3% of mothers and 6.1% of fathers reported a mean (SD) absence from work of 4.46 (2.11) and 4.31 (2.73) days, respectively, which were statistically significantly higher than those of influenza-negative patients [mothers: 12.0%, 3.39 (2.26), *p* < 0.05; fathers: 2.0%; 1.96 (2.04), *p* < 0.001]. This was significantly more evident among the parents of children aged <2 or 2–5 years than 5 years (*p* < 0.05) [39]. Similarly, Ploin et al., 2007, in France, found that 54% of caregivers of patients with influenza-positive status reported a mean (SD) absenteeism of 6.3 (4.7) days compared to 54% of caregivers in the influenza-negative group at 5.9 (4.3) days, although no *p*-values were reported in the study [24]. While Aykac et al., 2017, in Turkey, reported that 36% and 34% of fathers took leave for children with influenza-positive or -negative status, respectively (*p* = 1.0), without mentioning missed workdays. Meanwhile, no mothers were working in the influenza-positive group, and all 17 working mothers in the influenza-negative group took leave. The study also found that 71.4% of mothers, 60% of fathers, and 40% of both experienced sleep disturbances for children with influenza-positive status. 

##### Impact of Patients’ Influenza Viral Type (Influenza A or B) on Work Productivity of Caregivers, Europe 

Five studies examined the impact of influenza A (including A/H1N1 and A/H3N2) or influenza B on caregivers’ work productivity, involving a total of 5466 patients across 19 study cohorts (Figure 6D) [26,27,39,40,51]. The mean (SD) number of caregiver workdays missed varied across the different strains, including 1.2 (1.4) to 5.9 (2.6) days for A/H1N1, 2.8 (0.2) to 5.9 (2.6) days for A/H3N2, and 2.7 (0.2) to 3.0 (2.7) days for influenza B. Silvennoinen et al., 2015 reported that no statistically significant differences were observed between influenza A/H1N1, A/H3N2, and B infections in any age group [26]. Similarly, Streng et al., 2018 found no statistically significant difference among any group (*p* = 0.397) [27]. Meanwhile, Esposito 2011a reported statistically significantly higher missed days among parents of children with influenza A-positive vs. those of influenza B-positive cases (*p* < 0.05) [39], and Esposito et al., 2011b with a total sample size of 2547 across three seasons found statistically significantly higher missed workdays in the season of 2009-10 during the pandemic than seasonal influenza (mothers: *p* < 0.0001; fathers: *p* < 0.01) [40].

## 4. Discussion

This global systematic review synthesized studies published within the past 15 years assessing the impact of influenza or ILI on caregivers’ well-being, focusing on their HRQoL, work productivity, and activity impairment due to a patient’s illness. This review found that influenza/ILI continues to pose a significant burden extending beyond the affected patients, causing work productivity loss and HRQoL declines among informal caregivers. 

The available evidence for assessing the impact on HRQoL among caregivers of patients with influenza or ILI was limited, with only three identified studies focusing on caregivers of pediatric patients aged 6 months to up to 36 or 48 months with ILI. Among these, two studies employed a disease-specific instrument (Care-ILI-QoL), while one utilized Par-ENT-QoL, and all three incorporated SF-12v2. Despite the limited number, all three studies consistently demonstrated that caregivers experienced significantly lower QoL while their child had ILI compared to periods when their child did not have ILI, and/or in comparison to caregivers of children without ILI. The domains of emotions and social life were significantly affected, while a physical impact, in terms of daily life disturbance, was also notably observed. Moreover, the total QoL and factor scores derived from disease-specific instruments were found to correlate well with SF-12v2 scores in these studies. 

All three HRQoL studies highlighted a significant decline in QoL among caregivers who perceived their child as very/extremely sick compared to those perceiving their child as mildly sick, as well as among caregivers with longer durations of their child’s illness [7,17,18]. However, the lack of studies targeting different age groups and the relatively small sample sizes in cohorts, particularly for subgroup analyses, limited the generalizability of the findings. For instance, Chow et al., 2013b observed a similar QoL impact on caregivers for each ILI episode [18]. Nevertheless, only 16 pediatric patients reported experiencing more than one ILI episode, compared to 89 with just one ILI episode, suggesting that the comparable extent of QoL impact may be attributed to chance alone [18].

In contrast, a substantial body of evidence was identified across various regions, except Africa, examining the impact on work productivity among caregivers of patients with influenza or ILI. In the nine studies from the Asia–Pacific region (APAC), the average missed workdays ranged from 0.7 to 3.5 [19,20,22,30,31,37,42,47,48], which was comparatively lower than in American studies (0.5 to 9.1 days) [7,25,29,34,35,38,43,44,45] and European studies (1.2 to 10.7 days) [21,23,24,26,27,32,39,40,41,49,50,51]. However, these variations can largely be attributed to country-specific work policies, healthcare seeking behavior, social determinants of health, and cultural attitudes towards work and caregiving among developed regions like the US, the UK, Europe, and Australia, among others. These differences across dimensions such as work policies, wages and benefits, regulatory environments, and work culture can significantly impact employee rights, benefits, productivity levels, and sick leave policies within each region [53,54].

The proportion of caregivers affected by a patient’s illness, as well as the impact on their work productivity, varied widely depending on factors such as the influenza status (positive or negative), severity of the condition, age group, viral type, and vaccination status among patients with influenza or ILI. This pattern remained consistent across different regions, although only a few studies reported statistically significant results, and many studies did not report *p*-values. Notably, caregivers of hospitalized patients with influenza or ILI reported the highest numbers of missed workdays, with averages reaching up to 10.7 days in Spain. 

The reviewed studies covered influenza seasons from 1996 to 2022. Despite spanning a wide range of study periods, no clear trend was observed in the variability of reported workdays missed due to influenza or ILI across studies of different influenza seasons. This lack of a trend, however, does not necessarily imply that the outcomes (e.g., workdays missed) do not vary from season to season. Even if we did not observe them, specific trends in outcomes may be influenced by the multiple aforementioned factors that fluctuate independently of the chronological year and may relate to the severity of the types of strains circulating in specific seasons, transmission rates, levels of vaccine, and infection-induced uptake.

Most of the studies focused on caregivers of pediatric patients, particularly young children aged <5 years (n = 33) and those aged 5 to 12 years (n = 27), whereas only 15 studies included caregivers of adults aged 18 to <65 years, and seven studies addressed caregivers of elderly patients aged 65+ years. Most of the studies reported a wide range of missed workdays, making it difficult to interpret the results based on the age factor alone. However, there was no striking difference observed in workday loss among caregivers for young children compared to those of adults or the elderly, although caregivers with children had relatively higher rates of absenteeism. For instance, Fragaszy et al., 2017, in the UK, reported 1.6–2.9 missed workdays for caregivers of patients 0–15 years compared to 0.0–2.0 days for those of adults 16–64 years and 0.0–2.5 days for caregivers of patients 65+ years, with absenteeism rates of 24–70%, 0–10%, and 0–6%, respectively [33].

Some studies noted that primary caretakers, such as mothers, were not employed, and thus work productivity was not accounted for in those cases. Moreover, only three studies reported data on presenteeism, or the impact on leisure or unpaid activities among caregivers—aspects that can be significantly affected by a patient’s illness. Consequently, while there is significant evidence on the number of missed workdays among caregivers, the true extent of the burden remains difficult to quantify due to variations in reporting and the limited scope of the studies. This underscores the need for more comprehensive research to capture the full impact on caregivers’ productivity and well-being.

To our knowledge, this is the first systematic review exclusively focusing on the caregiver burden resulting from a family member’s influenza or ILI. Zumofen et al., 2023 conducted a systematic review to assess the impact on work productivity due to influenza or ILI, covering both patients and caregivers. The authors included 17 caregiver-specific studies with 31 study cohorts, reporting a mean caregiver absenteeism of 1–2 days, ranging from 0.5 days to 9.1 days [6]. In contrast, our review identified 34 studies with more than 120 study cohorts (including those captured by Zumofen et al., 2023 [6]), with a mean absenteeism ranging from 0.5 days to 10.7 days. Our review complements this previously published work by not only identifying nearly twice as many studies on work productivity studies but also conducting a comprehensive search to assess the caregivers’ humanistic burden on HRQoL. Furthermore, we synthesized studies by geographic region as well as by key factors such as influenza status, viral type, severity, and patient age group, wherever data were available, covering 27 influenza seasons from 1996 to 2022. 

However, it is important to acknowledge certain limitations when interpreting the findings of this review. Despite employing a broad search strategy and including multiple databases, some of the relevant literature, particularly that available through alternative sources and in languages other than English, may not have been captured in our review.

While the focus of this review was to assess caregiver-related burden, most identified studies on work productivity outcomes predominantly assessed overall disease burden for patients and caregivers, without a specific or sole emphasis on caregivers. Only 47% of the 34 studies provided caregiver-related demographic details or other information. The studies that reported caregiver-related details were also limited and varied widely in terms of included variables. The reviewed studies mainly reported work productivity outcomes in terms of the percentage of caregivers missing work and the mean or median number of workdays lost due to their children or household member’s influenza or ILI. However, 41% of the studies did not explicitly report the number of caregivers and/or the percentage of caregivers reporting absenteeism in the overall study or sub-cohort. As a result, we used the number of patients as a proxy if the number of caregivers was missing, assuming each patient was accompanied by one caregiver. Additionally, due to the significant heterogeneity observed in study designs, populations, case definitions, and outcomes, it was not feasible to aggregate nor perform a comparison of the caregiver outcomes across studies. Hence, we narratively synthesized the data as reported by the study authors and did not perform a feasibility assessment for conducting a meta-analysis, which could have potentially offered deeper insights into the impacts on work productivity and quality of life among caregivers. The selected studies in the review were primarily observational studies, which are susceptible to various sources of bias such as selection bias, information bias, and recall bias, as well as errors and omissions. However, due to the aforementioned data limitations, a risk of bias assessment was not performed. The limitations reported by the authors were reviewed and considered when evaluating and interpreting the whole body of evidence.

Our review focused exclusively on the burden among informal caregivers due to their family members or friends’ influenza or ILI, without assessing the burden among patients, formal caregivers such healthcare facilities or secondary infections among caregivers, and their potential effects. Consequently, the burden reported in our review may underestimate the actual impact of influenza or ILI on caregivers.

Although we excluded studies or subgroups reporting impacts on caregivers due to their own illnesses, it was challenging to determine whether the caregivers had prior influenza or ILI because most of the studies did not exclusively describe caregivers’ related information. Other studies have examined the impact on work productivity and caregiving due to the caregivers’ own influenza or ILI [43,55]. 

For example, Waite et al., 2022 conducted surveys of Canadian adults aged 50 and older to assess the impact of influenza or ILI on working, volunteering, and caregiving using the EXamining the Knowledge, Attitudes, and Experiences of Canadian Seniors Towards Influenza (EXACT) questionnaire [55]. They surveyed 1006 adults aged 50–64 years during the 2018–19 influenza season and 1001 during the 2019–20 season, as well as 3458 and 3500 individuals aged 65 and older during the 2018–19 and 2019–20 seasons, respectively. Among those aged 50–64 years with influenza or ILI, 97 (42.2%) were caregivers in 2018–19, and 57 (22.2%) in 2019–20. In 2018–19, 41.2% were unable to provide care while ill for a mean of 5.3 days, and 45.4% provided care at a reduced capacity (mean = 6.4 days). In comparison, in 2019–20, 49.1% were unable to provide care for a mean of 6.9 days, and 47.4% provided care at a reduced capacity for a mean of 6.1 days. In the 65+ age group, 123 (24.6%) were caregivers in 2018–19, and 162 (27.0%) in 2019–20. In 2018–19, 14.6% were unable to provide care while ill (mean  =  7.8 days), compared to 25.9% in 2019–20 (mean  =  13.1 days, *p* < 0.02). Additionally, 42.3% of individuals provided care at a reduced capacity in 2018–19, compared to 28.4% of individuals in 2019–20 (mean  =  5.8 vs. 8.8 days; *p* < 0.01). These findings highlight the significant impact of influenza or ILI on caregivers, including both direct effects on their ability to provide care and broader implications for work productivity. Future studies should consider such factors, including the infection status of caregivers and the transmissibility of disease, to capture the comprehensive burden of influenza or ILI more accurately. 

We included only quantitative studies for HRQoL and work productivity outcomes, which could potentially be used in cost effectiveness modeling for health interventions. We excluded qualitative studies that could also provide valuable insights into the overall caregiver burden.

Additionally, studies from various countries assessed different outcomes using various measurement methods including surveys, interviews, and structured questionnaires. However, none of the studies reported using standard validated tools like the WPAI [46]. Consequently, we did not include cost components related to work absenteeism to estimate productivity loss in monetary terms, as wages may differ substantially across countries by age, gender, profession, work experience, and other factors.

We also categorized the identified studies into different geographic regions based on the study settings and locations; however, most of the studies were cross-sectional or cohort studies with limited sample sizes. Therefore, the estimated work productivity loss reported in these studies might not accurately represent the national or regional burden among caregivers due to influenza or ILI. 

## 5. Conclusions

This systematic review complements prior work to show that the consequences of influenza and ILI extend far beyond the immediate health impacts on patients. Influenza or ILI significantly affect caregivers and result in substantial work productivity loss, which can generate indirect costs and societal burden. Caregivers often experience considerable work productivity losses, including both absenteeism and presenteeism, as well as disruptions to their daily routines, leisure activities, and unpaid activities. These impacts contribute to a broader economic strain on families and communities. Additionally, the review highlights the notable humanistic burden among caregivers, including adverse effects on their physical and mental health, leading to a reduced quality of life. These findings help to characterize the broad societal burden of influenza, which goes beyond the affected patients. They may raise awareness of the unmet needs related to influenza and the magnitude of spillover effects. Different stakeholders, such as healthcare providers and employers, may consider utilizing these findings to inform continued efforts to manage and address the multifaceted impacts of influenza and ILI. Additionally, the quantitative estimates obtained from this review could be used to quantify the health economic value of health interventions targeted for influenza.

## Figures and Tables

**Table 1:** Impact on the health-related quality of life (HRQoL) among caregivers of patients with influenza or influenza-like illness (ILI).

Study, Year	PRO Instrument	Study Cohort, Number of Caregivers (N)	Impact on the Health-Related Quality of Life among Caregivers of Patients with Influenza or Influenza-like Illness (ILI)					
			Daily Activities Disturbance	Perceived Support	Social Functioning	Emotional Domain	Overall QoL	
			Mean (SD)	Mean (SD)	Mean (SD)	Mean (SD)	Mean (SD)	Median [Q1–Q3]
Chow 2013a [17]	Care-ILI-QoL (1: lowest QoL; 7: highest QoL)	ILI, 55	3.36 (1.41)	4.86 (1.47)	3.24 (0.84)	4.00 (1.30)	3.87 (0.93)	3.81 range: [1.00–5.83]
Chow 2013b [18]	PAR-ENT-QoL (0: lowest QoL; 100: highest QoL)	Baseline, ILI, 105	84.84	Not Applicable		74.91	79.77	Not Reported
		Post-ILI interviews, ILI, 105	61.91			60.22	60.99	
		Baseline, non-ILI, 276	85.82			78.58	82.3	
		Post-ILI interviews, non-ILI, 276	86.81			81.28	84.05	
Overmann 2023 [7]	Care-ILI-QoL (1: lowest QoL; 7: highest QoL)	ILI, 281	3.70 (1.54)	5.20 (1.88)	3.70 (1.30)	2.80 (1.58)	3.90 (1.52)	4.00 [3.00, 4.00]

Care-ILI-QoL, quality of life of caregivers of children with influenza-like-illness; ILI, influenza-like-illness; PAR-ENT-QoL, Parent’s Quality of Life as related to Ear, Nose, and Throat; PRO, patient-reported outcome; QoL, quality of life; Q1–Q3, first and third quartiles; QoL, quality of life; SD, standard deviation. Note—Perceived support in the Care-ILI-QoL was defined using three items that assessed how satisfied caregivers were with practical and emotional support from their immediate or extended family, as well as emotional support from friends.

## Data Availability

The original contributions presented in the study are included in the article/Supplementary Materials, further inquiries can be directed to the corresponding author.

## Supplementary Materials

The following supporting information can be downloaded at: https://www.mdpi.com/article/10.3390/healthcare12161591/s1, Table S1: PICOS Criteria; Table S2: Search Strategy; Table S3: Example, Database Search Strategy and Results, MEDLINE via PubMed; Table S4: Database Search Results; Table S5: Study Characteristics, Health-Related Quality of Life (HRQoL); Table S6: Caregiver-specific Study Characteristics, Health-Related Quality of Life (HRQoL); Table S7: Study Characteristics, Work Productivity; Table S8: Caregiver-specific Study Characteristics, Work Productivity; Table S9: Results, Work Productivity.
