# Supplementary material for: Caregiver Burden among Patients with Influenza or Influenza-like Illness (ILI): A Systematic Literature Review
Source: Healthcare (Basel). 2024 Aug 9;12(16):1591. doi: 10.3390/healthcare12161591 (PMC11353737; doi:10.3390/healthcare12161591)
Supplement: Supplementary file 1 [file healthcare-12-01591-s001.zip › healthcare-3062397-supplementary.pdf]

Supplemantary Materials: Caregiver Burden among Patients with Influenza or Influenza-Like Illness (ILI): A Systematic Literature Review

Table S1. PICOS Criteria.

	Criteria	Inclusion	Exclusion
**PICO(S)**	Population/ participants	Informal caregivers of patients with influenza or influenza-like illness (ILI)	Animal studies, non influenza/ILI studies
	Interventions	Any	N/A
	Comparators	Any	N/A
	Outcomes	Impacts on caregivers' health-related quality of life (HRQoL) and productivity measures due to patient's illness:<br>1. HRQoL (e.g., health disutilities related to physical, psychological with social functioning and well-being)<br>2. Work productivity measures (e.g., absenteeism, presenteeism, work impairment, and time burden)	- Studies assessing the impact of outbreak/pandemic restrictions (e.g., outbreak school closures) on caregivers and their HRQoL or work productivity.<br>- Studies assessing outcomes due to caregiver's own influenza/ILI illness (secondary infection).
	Study design/Setting	All study designs based on empirical research (i.e., randomized or non-randomized studies including observational studies).	- Non-research publications (editorials, reviews)<br>- Modeled outcomes.<br>- Prior SLRs will be screened to identify studies, but no direct data extraction will be performed from those reviews.
**Other eligibility criteria**	Geographical location	No restriction by location	NA
	Study period	Published from January 1, 2007, to April 30 2024	Before 2007
	Language criteria	English	Any other languages

Table S2. Search Strategy.

#	Search Terms
**S1**	Influenza OR influenza-like illness
**S2**	parents OR caregivers OR mothers OR father OR spouse OR family OR household
**Work Productivity**	**S1 AND S2**<br>**AND**<br>("work performance" OR "work productivity loss" OR "work presenteeism" OR "work impairment" OR "work effect" OR "work performance" OR "work efficiency" OR "productivity loss" OR "lost earn" OR "work absence" OR "absence from work" OR "time off work" OR "absenteeism" OR "work absenteeism" OR "sickness absence" OR "return to work" OR "sick leave" OR "sick day" OR "productivity" OR "presenteeism" OR "indirect cost")
**Health-related Quality of Life (HRQoL)**	**S1 AND S2**<br>**AND**<br>("quality of life" OR utilities OR "disutilities" OR "EQ 5D" OR "EQ-5D" OR "quality adjusted life year" OR "QALY" OR "activities of Daily Living" OR "daily living" OR "HUI" OR "Health Utility Index")

Table S3. Example, Database Search Strategy and Results, MEDLINE via PubMed.

#	Search Terms	Results
1	Influenza* OR flu* OR influenza-like-illness*	167,682
2	"parents"[TIAB] OR "caregivers"[TIAB] OR "mothers"[TIAB] OR "father"[TIAB] OR "spouse"[TIAB] OR "family"[TIAB] OR "household"[TIAB]	446,327
3	#1 and 2	2,314
4	("work performance"[TIAB] OR "work productivity loss"[TIAB] OR "work presen*"[TIAB] OR "work product*"[TIAB] OR "work impair*"[TIAB] OR "work effect*"[TIAB] OR "work perform*"[TIAB] OR "work efficiency*"[TIAB] OR "productivity loss"[TIAB] OR "lost earn*"[TIAB] OR "work absence*"[TIAB] OR "absence from work"[TIAB] OR "time off work"[TIAB] OR "absenteeism"[TIAB] OR "work absenteeism"[TIAB] OR "sickness absence"[TIAB] OR "return to work"[TIAB] OR "sick leave"[TIAB] OR "sick day"[TIAB] OR "productivity"[TIAB] OR "presenteeism"[TIAB] OR "indirect cost"[TIAB])	130,612
5	("quality of life"[TIAB] OR "utilit*"[TIAB] OR "disutilit*"[TIAB] OR "EQ 5D"[TIAB] OR "EQ-5D"[TIAB] OR "quality adjusted life year"[TIAB] OR	4,262,383

	"QALY"[TIAB] OR "activit*"[TIAB] OR "activities of Daily Living"[TIAB] OR "daily living"[TIAB] OR "HUI"[TIAB] OR "Health Utility Index"[TIAB])	
6	# 3 and 4	63
7	Filters: Humans; Jan 1, 2007, to April 30, 2024; English	54
8	#3 and 5	154
9	Filters: Humans; Jan 1, 2007, to April 30, 2024; English	96

Table S4. Database Search Results.

#	Database	Health-related Quality of Life (HRQoL)	Work Productivity	**Total**
1	PubMed	55	96	151
2	APA PsycNet	633	1515	2148
3	Cochrane	268	172	440
4	Embase	4381	1928	6309
5	Ovid	3560	2315	5875
6	Web of Science	2278	1228	3506
**7**	Hand search	125	135	260
	**Total**	11,300	7389	18,689

Table S5. Study Characteristics, Health-Related Quality of Life (HRQoL).

Study	Country	Study Design	Study Period	Patient Population	Patient Disease Definition	Study setting	PRO Instrument	Instrument Details
Chow 2013a [1]	Australia	RCT	Influenza season: 2011	Children aged 6–48 months	ILI was defined as fever ≥37.8 °C or feverishness plus runny nose or sore throat or cough	48 childcare centres in Sydney, Australia	Care-ILI-QoL<br><br>SF12v2 Acute Form	Care-QoL-ILI—16-item disease-specific measure covering four domains: daily activities, perceived support, social life, and emotions.<br>- 7-point Likert scale with a recall period of past 7 days for each question (e.g., "much worsened," "stayed the same," and "much improved") allowing for subjective comparison across time.<br>- It also measures caregiver perception of disease severity.<br>- Care-QoL-ILI scores were calculated for each domain, as well in aggregate, with 7 being the highest (best) possible QoL and 1 being the lowest (worst).<br><br>Medical Outcomes Study Short-Form version 2 (SF-12v2) - A generic assessment of HRQoL of the caregivers themselves, allowing comparison of baseline differences that may affect acute changes in QoL during the ILI.
Chow 2013b [2]	Australia	Prospective cohort	Influenza Season: Before Influenza: Mar to Jul 2010 During Influenza: Jul to Nov 2010	Children aged 6 months–3 years	ILI was defined as fever ≥37.8 °C or feverishness according to the caregiver's judgement, plus runny nose, or sore throat or cough	90 childcare centres and one paediatric-focused GP practice in Sydney, Australia	PAR-ENT-QoL<br><br>SF-12v2 Acute Form	The PAR-ENT-QoL: 15-item questionnaire covering two main domains: emotional and daily disturbance<br>- 15 items regarding the extent of parents' worry, stress, mood, quality of sleep, time for family members, outings and leisure, medical expenses, and perceived QoL in general during the preceding 2 weeks.

								- 5-point Likert scale with a recall period of 2 weeks<br>- The domain scores were calculated by adding up the scores (1–5) of items from the corresponding domain. The domain scores were then linearly transformed (ranging from 0 to 100).<br>- The Total score was calculated by adding up the two domain scores and dividing by two.
Overmann 2023 [3]	USA	Cross-sectional	Enrollment: Feb 2020 to May 2021	Pediatric patients aged 6 to 48 months with study site measured/caregiver-reported ILI	ILI was defined as an acute respiratory disease of sudden onset and fever (temperature ≥38 °C) measured at the study site or reported by caregivers within 24 h prior to presentation and accompanied by at least one of the symptoms of cough, nasal congestion, runny nose, or sore throat.	Pediatric ED and UC at Cincinnati Children's Hospital Medical Center (main hospital site and a suburban satellite location)	Care-ILI-QoL<br><br>SF-12v2	Used Care-ILI-QoL referring Chow 2013a during the study (Description as above).

Abbreviations: Care-ILI-QoL, quality of life of caregivers of children with influenza-like-illness; ILI, influenza-like-illness; PAR-ENT-QoL, Parent's Quality of Life as related to Ear, Nose, and Throat; PRO, patient-reported outcome; SD, standard deviation; SF-12v2; Short Form Health Survey version 2

Table S6. Caregiver-specific Study Characteristics, Health-Related Quality of Life (HRQoL).

Study	Country	Study Cohort Population, N	Caregiver-specific details
Chow 2013a [1]	Australia	ILI 55	**Caregivers of children aged mean (SD) 27 (12) and range (8-49) months with ILI; N=55**<br><br>**Caregivers age:** range (24.1-41.7); mean (SD) 33.7 (3.9) yrs.<br>**Relationship to child n (%):** Mothers: 52 (95%); Fathers: 3 (5%)<br>**Caregiver education level n (%):** High school: 5 (9%); vocational diploma: 9 (16%); university and above: 41 (75%)<br>**Marital status n (%):** Married: 49 (89%); De facto: 4 (7%); Divorced/Separated/Widowed: 2 (2%)<br>**Gross annual household income (USD):** Range: 34,666-235,237; Mean: 123,972; SD: 52,759<br>**Working outside home:** Yes: 36 (66%); No: 12 (22%); Maternity leave 7 (13%)
Chow 2013b [2]	Australia	Baseline, ILI, 105<br>Post-ILI interviews, ILI, 105<br>Baseline, non-ILI, 276<br>Post-ILI interviews, non-ILI, 276	**Parents of children aged mean (SD) 26 (8.5) and range (11-40) months who had ILI; N=105**<br><br>**Parents, ILI**<br>**Parents age** mean (SD) 36.0 (4.1) and range 26-51 yrs.<br>**Relationship to child n (%):** Mothers 100 (95%); Fathers 5 (5%)<br>**Parents 'education level n (%):** Below University: 21(20%); University or above: 83 (79%); Missing: 1 (1%)<br>**Weekly household income (AUD) n (%):** Below < $1,000: 1(1%); $1,000–$1,999: 19 (18%); $2,000–$2,999: 59 (56%); $3,000 or above: 22 (21%); Missing 4 (4%)<br>Household employment status: Both parents working:79 (75%); Mother on maternal leave: 6 (6%); Only one parent working: 19 (18%); Both parents not working: 0 (0); Missing: 1 (1%)<br><br>**Parents of children aged mean (SD) 28 (7.7) and range (11-41) months who had ILI; N=276**<br><br>**Parents, non-ILI**<br>**Parents age:** mean (SD) 36.0 (4.3) and range 21-51 yrs.<br>**Relationship to child n (%):** Mothers: 261 (95%); Fathers: 15 (5%)<br>**Parents 'education level n (%):** Below University: 89 (32%); University or above: 186 (67%); Missing 1 (0.4%)<br>**Weekly household income (AUD):** Below < $1,000: 9 (3%); $1,000–$1,999: 57 (21%); $2,000–$2,999: 123 (45%); $3,000 or above: 60 (22%); Missing 27 (10%)<br>**Household employment status:** Both parents working: 199 (72%); Mother on maternal leave: 12 (4%); Only one parent working: 62 (23%); Both parents not working: 3 (1%); Missing: 0 (0%)

Overmann 2023 [3]	USA	ILI, 281	**Caregivers of children aged mean (SD) 6.3 (2.1) and median 5.4 (1-8) yrs with ILI; N=281**<br><br>**Caregiver age n (%):** <20yrs: 7 (2.5%); 20-24yrs: 65 (23.1%); 25-29yrs: 75 (26.7%); 30-34yrs: 75 (26.7%); 35-39yrs: 42 (14.9%); 40-44yrs: 9 (3.2%); ≥45yrs: 1 (0.4%); Unspecified: 7 (2.5%)<br>**Relationship to child n (%):** Mother:264 (93.9%); Father: 14 (5.0%); Grandmother: 3 (1.1%)<br>**Caregiver highest education level n (%):** Less than high school graduate: 18 (6.4%); High school graduate: 78 (27.8%); Vocation/tech training school: 12 (4.2%); Some college: 73 (26.0%); College graduate: 68 (24.2%); Postgraduate: 32 (11.4%)<br>**Household income (USD) n (%):** Less than $5000: 57 (20.3%); $5001 to $15 000: 28 (10.0%); $15 001 to $30 000: 54 (19.2%); $30 001 to $50 000: 47 (16.7%); $50 001 to $75 000: 24 (8.5%); $75 001 to $90 000: 17 (5.7%); $90 001 to $119 999: 24 (8.5%); More than $120 000: 27 (9.6%); Not reported: 3 (1.1%)<br>**Caregiver employment Status n (%):** Employed: 181 (64.4%); Full time: 127 (45.2%); Part time: 54 (19.2%)

Table S7. Study Characteristics, Work Productivity.

Study Details				Patient Population (Patients with influenza/ILI)		Study Setting	Outcomes of Interest	Measurement Method/ Instrument
Study, Year	Location	Study Design	Study period/Influenza Seasons	Patient Population	Diagnosis Type			
Overmann 2023 [3]	USA	Cross-sectional	Enrollment: February 17, 2020 - May 7, 2021	Age, patient with ILI: mean (SD) 6.34 (2.1); median 5.4 (1-8)	Study site measured/self-reported ILI	Pediatric ED and UC at Cincinnati Children's Hospital Medical Center (main hospital site and a suburban satellite location)	Workdays missed	Questionnaire
Romanelli 2023 [4]	United Kingdom	Cross-sectional	Four influenza seasons of Autumn 2018 to Spring 2022	A geographically representative survey of 1,000 working-age adults (aged 18–64) who reported having influenza or caring for a dependent with influenza during at least one of the past four influenza seasons (from Autumn 2018 to Spring 2022).	Self-reported	Geographically representative survey in UK	Days off work	Survey
Zhang 2022 [5]	China	Cross-sectional	Influenza season: 2017-2018	Children with aged under 5 years	Self-reported ILI	Internet survey in six provinces, including Henan, Shandong, and Tianjin in northern China, and Guangdong, Guangxi, and Sichuan in southern China	Absenteeism among family members of children with ILI: N (%) and Median, Day (IQR)	Online questionnaire
Lai 2021 [6]	China	Cross-sectional	Influenza season: 2018–2019	Children aged 6–59 months;<br>Chronic disease patients aged 18–59 years;<br>Elderly aged 60+ years;	Self-reported ILI	148 community health centers from 10 provinces in China	Lost labor days of respondents' families	Structured online questionnaire

Study Details				Patient Population (Patients with influenza/ILI)		Study Setting	Outcomes of Interest	Measurement Method/ Instrument
Study, Year	Location	Study Design	Study period/Influenza Seasons	Patient Population	Diagnosis Type			
Wang 2021 [7]	China	Prospective observational	Influenza seasons: 2011-2012 to 2016-2017	Children < 5-year-old with laboratory-confirmed influenza/ILI	Laboratory	Surveillance in Suzhou during the influenza seasons	Lost productivity days of caregivers, median days (IQR) for outpatients and inpatients	Data from prospective community-based cohort studies and hospital-based surveillance
Rao 2020 [8]	USA	Prospective observational	Enrollment: January to April 2017 and from November 2017 to April 2018.	Children 6 months–8 years of age presenting with influenza-like illness (ILI)	Laboratory	Children's Hospital Colorado (CHCO) ED and an affiliated urgent care (UC) center	Parent absenteeism, d, median (IQR)	Interviews
Salcedo-Mejía 2019 [9]	Colombia	Retrospective observational	January and August 2014	Pediatric patients (<18 years old) with influenza-confirmed severe acute respiratory infection	Laboratory	A university hospital (Fundación Hospital Infantil Napoleón Franco Pareja) in the city of Cartagena.	Number of days absent from work	Questionnaire
Willis 2019 [10]	Australia	Cross-sectional	Influenza seasons 2008-2014	Young children eligible for vaccination (aged 6-59 months at the time of vaccination) presenting to the emergency department (ED) or admitted with ILI	Laboratory	Princess Margaret Hospital, Perth WA	Parent Work Absenteeism (hours)	Questionnaires
Streng 2018 [11]	Germany	Prospective observational	Influenza season: 2013 to 2015 (January–May)	Children 1–5 years of age, unvaccinated against influenza and presenting with febrile acute respiratory infections (ARIs)	Laboratory	33 outpatient pediatric practices in Bavaria, Southern Germany.	Parent Workdays lost after practice visit, median (IQR)	Questionnaire
Aykac 2017 [12]	Turkey	Prospective observational	December 2015 and April 2016	Children aged <18 years with symptoms of influenza infection	Laboratory	Hacettepe University Ihsan Dogramaci Children's Hospital	Productivity loss of caregivers<br>Sleep disturbance among caregivers	Comfort assessment

Study Details				Patient Population (Patients with influenza/ILI)		Study Setting	Outcomes of Interest	Measurement Method/ Instrument
Study, Year	Location	Study Design	Study period/Influenza Seasons	Patient Population	Diagnosis Type			
Thorrington 2017 [13]	United Kingdom	Retrospective observational	Influenza seasons 2012-2013 and 2013-2014	School children with symptoms consistent with ILI	Caregiver/self-reported	primary schools in England	Mean number of caregivers used during absence (95% CI)<br>Mean total time off work for all caregivers (95% CI)	Paper-based and online questionnaires
Fragaszy 2017 [14]	United Kingdom	Prospective cohort	6 influenza seasons including 3 periods of seasonal influenza (winters 2006-2007, 2007-2008 and 2008-2009) and the first 3 waves of the 2009 influenza pandemic (summer 2009, autumn-winter 2009/2010 and winter 2010/2011)	Individuals randomly recruited through primary care practices	Laboratory	Primary care practices, England	Work productivity	Survey
Heikkinen 2016 [15]	Finland	Prospective cohort	Study 1: Two consecutive winter seasons of 2000–2001 and 2001–2002<br><br>Study 2: Influenza seasons of 2007–2008 and 2008–2009	Study 1: Children ≤13 years of age, with 2,231 child-seasons of follow-up<br>Study 2: Children 1–3 years of age; with 1,185 children, 764 of whom were assessed for respiratory symptoms	Laboratory	Outpatient children in Turku, Finland.	Parental work absenteeism- Mean duration of absence (days), Total days of absence per 100 children	Symptom diaries and questionnaire

Study Details				Patient Population (Patients with influenza/ILI)		Study Setting	Outcomes of Interest	Measurement Method/ Instrument
Study, Year	Location	Study Design	Study period/Influenza Seasons	Patient Population	Diagnosis Type			
Mughini-Gras 2016 [16]	The Netherlands	Cross-sectional	October 2012-October 2014	Households with children younger than 4 years	Self-reported	Households randomly selected from population registries of 335 (out of 415) municipalities in the Netherlands	Work absenteeism (days) median, IQR	Web-based questionnaire
Tinoco 2015 [17]	Peru	Community-based cohort	June 2009–December 2010	2,000 randomly selected households comprising 7,200 people	Laboratory	Four geographically diverse sites in Peru	Absenteeism among caregivers as well as hours in unpaid activities	Standardized Questionnaire
Silvennoinen 2015 [18]	Finland	Prospective observational	2 consecutive winter seasons (October–May, 2000–2002)	Pre-enrolled cohorts of children ≤13 years of age	Laboratory	Structured medical records filled out by study physicians	Parental work absenteeism:<br>Absent for ≥1 day, No. (%)<br>Mean duration of absence, days (SE)<br>Total days of absence per 100 children (95% CI)	Symptom diaries and questionnaire
Enserink 2014 [19]	The Netherlands	Cross-sectional	October 2012 to October 2013	Households with children aged 0–48 months	Self-reported ILI	2000 children - one child per household – were randomly selected from the population registries of 415 Dutch municipalities.	Work absenteeism	Survey
Silva 2014 [20]	France	Prospective observational	Influenza season: 2010-2011	Patients with lab-confirmed Influenza B	Laboratory	all practitioners participating in the GROG network (Groupes Régionaux d'Observation de la Grippe), which included 390 general practitioners (GPs) and 116 pediatricians, France	Work productivity	Study forms

Study Details				Patient Population (Patients with influenza/ILI)		Study Setting	Outcomes of Interest	Measurement Method/ Instrument
Study, Year	Location	Study Design	Study period/Influenza Seasons	Patient Population	Diagnosis Type			
Bhuiyan 2014 [21]	Bangladesh	Prospective observational	Influenza season: May to October, 2010	Inpatients and outpatients from the influenza surveillance program database	Laboratory	A sentinel influenza surveillance program at one private and three government tertiary hospitals in four districts of Bangladesh, Kishorgonj, Comilla, Bogra, and Barisal	Productivity loss of caregiver: median (IQR) workdays, and associated indirect costs (US$)	Structured questionnaire
Wang 2013 [22]	China	Prospective observational	March 2011-February 2012	Children <5 years old and had symptoms of ILI defined as the presence of fever (axillary temperature ≥38°C) and cough or sore throat	Laboratory	Soochow University Affiliated Children's Hospital (SCH) in Jiangsu province, China.	Lost parental workdays, mean ± SD Parental loss of earnings	Telephone survey
Yin 2013 [23]	Australia	Prospective cohort	Influenza season: January 2010 to March 2010	Children aged ≥6 months to <3 years with laboratory confirmed ILI	Laboratory	90 childcare centres and one general practitioner clinics in Sydney, Australia.	Work time lost (hours), Leisure time lost (hours), Cost of Work time lost	Telephone interview
Ambrose & Antonova 2013 [24]	Study 1: Belgium, Finland, Israel, Spain, UK<br><br>Study 2: Belgium, the Czech Republic, Finland, Germany, Israel, Italy, Poland, Spain, Switzerland, UK<br><br>Study 3: Belgium,	Prospective cohort of three RCTs	Study 1: Two consecutive influenza seasons, between October 2, 2000, and May 31, 2002.<br><br>Study 2: 2002–2003 influenza season<br><br>Study 3: 2002–2003 influenza season	Participants with live attenuated influenza vaccine (LAIV) versus placebo or inactivated influenza vaccine (IIV):<br><br>Study 1: Children 6–35 months of age attending day care<br><br>Study 2: Children 24–71 months of age with recurrent respiratory tract infections<br><br>Study 3: Children 6–17 years of age with asthma	Laboratory	RCTs across Europe, UK and Israel	Parental work loss (days)	Data collected from three RCTs

Study Details				Patient Population (Patients with influenza/ILI)		Study Setting	Outcomes of Interest	Measurement Method/ Instrument
Study, Year	Location	Study Design	Study period/Influenza Seasons	Patient Population	Diagnosis Type			
	Finland, Germany, Greece, Israel, Italy, the Netherlands, Norway, Poland, Portugal, Spain, Switzerland, UK							
Galante 2012 [25]	Spain	Prospective observational	Influenza season: 2009 to March 2010	Hospitalized cases older than 6 months with confirmed influenza A (H1N1) 2009	Laboratory	36 hospitals of seven Spanish Autonomous Communities (Spain)	Work Absenteeism (day)	Telephone interview
Chiu 2012 [26]	China	Prospective observational	October 2003 to September 2006	All patients <18 years with a Hong Kong Island home address admitted to PYNEH or QMH for asthma exacerbation with or without fever, or a febrile acute respiratory infection defined as fever ≥38 ◦C with any respiratory symptom such as cough, runny nose or sore throat	Laboratory	PYNEH and QMH hospitals, Hong Kong	Parental work loss (days)	Questionnaire
Ortega-Sanchez 2012 [27]	USA	Prospective observational	Influenza season 2003-2004	Children aged <5 years with laboratory-confirmed, medically attended influenza	Laboratory	Three county children - Monroe County, New York (Rochester), Davidson County, Tennessee (Nashville), and Hamilton County, Ohio (Cincinnati), who were admitted with a diagnosis of acute respiratory tract infection and/or fever were enrolled	Caregivers time off (h)<br>Caregivers time costs ($)	Telephone interview

Study Details				Patient Population (Patients with influenza/ILI)		Study Setting	Outcomes of Interest	Measurement Method/ Instrument
Study, Year	Location	Study Design	Study period/Influenza Seasons	Patient Population	Diagnosis Type			
Esposito 2011a [28]	Italy	Prospective observational	Influenza seasons 1 November 2008 and 30 April 2009	Patients aged less than 14 years without any underlying chronic severe disease who were examined by the participating PCPs because of signs and/or symptoms of ILI.	Laboratory	Primary care pediatricians (PCPs) working for the National Health Service in the community in Italy	Mothers/Fathers who remained absent from work:<br>No. (%)<br>Working days lost, mean days ±SD	Telephone interview
Esposito 2011b [29]	Italy	Prospective observational	Influenza seasons 2007-2010	Patients aged less than 15 years without any underlying chronic severe disease who attended the ER or were hospitalised for an influenza like illness	Laboratory	Department of Maternal and Paediatric Sciences of the<br>University of Milan	Working days lost by mothers,<br>mean ± SD, working days lost by fathers, mean ± SD	Interviews using standardised questionnaires
Heinonen 2010 [30]	Finland	RCT	Influenza season:<br>2007–2008<br>2008–2009	Children 1–3 years of age with laboratory-confirmed influenza	Laboratory	Single primary care clinic RCT in Turku, Finland.	Absence from work	Symptom diaries and questionnaire
Palmer 2010 [31]	USA	Prospective observational	Influenza season: 2007-2008	Employees with Household members'/child's ILI	Self-reported	Employees with household ILI, child household ILI	ILI related absenteeism and presenteeism	Survey
Iskander 2009 [32]	Australia	Prospective observational	June 1-October 31, 2006	Children <5 years hospitalised with ILI (fever and respiratory symptoms).	Laboratory	The Children's Hospital at Westmead (CHW), a secondary and tertiary referral paediatric teaching hospital servicing New South Wales, Australia	Parents' work absenteeism (days)	Telephone interview
Bourgeois 2009 [33]	USA	Prospective observational	2 influenza and RSV seasons—December 2003 to April 2004 and November 2004 to May 2005	Children aged ≤7 years and treated in the ED of a tertiary care pediatric hospital for an acute respiratory infection	Laboratory	ED of a tertiary care pediatric hospital in Massachusetts	Workday missed by caregiver	Standardized interview

Study Details				Patient Population (Patients with influenza/ILI)		Study Setting	Outcomes of Interest	Measurement Method/ Instrument
**Study, Year**	**Location**	**Study Design**	**Study period/Influenza Seasons**	**Patient Population**	**Diagnosis Type**			
Johnson 2008 [34]	USA	Retrospective observational	November 2-12, 2006	Household	Self-reported	Yancey County residents with children in the public school system	Missed workdays	Telephone interview
Li 2007 [35]	USA	Retrospective observational	1996-2002	Healthy households with children (5–17 years)	Self-reported	Household US survey	Missed workdays	Health Survey
Ploin 2007 [36]	France	Prospective cohort	Influenza seasons of 2001-2002	Febrile children younger than 36 months presenting with fever onset (38°C or more) within 4 days of onset	Laboratory	pediatric emergency department of Edouard Herriot university hospital (Lyon, France)	Parental absenteeism	Structured questionnaire

Table S8. Caregiver-specific Study Characteristics, Work Productivity.

Study Details		Study Cohort Population, N	Caregiver-specific details
**Study, Year**	**Location**		
Overmann 2023 [3]	USA	281 caregivers	**Caregiver age n (%):** <20yrs: 7 (2.5%); 20-24yrs: 65 (23.1%); 25-29yrs: 75 (26.7%); 30-34yrs: 75 (26.7%); 35-39yrs: 42 (14.9%); 40-44yrs: 9 (3.2%); ≥45yrs: 1 (0.4%); Unspecified: 7 (2.5%)<br><br>**Relationship to child n (%):** Mother:264 (93.9%); Father: 14 (5.0%); Grandmother: 3 (1.1%)<br><br>**Caregiver highest education level n (%):** Less than high school graduate: 18 (6.4%); High school graduate: 78 (27.8%); Vocation/tech training school: 12 (4.2%); Some college: 73 (26.0%); College graduate: 68 (24.2%); Postgraduate: 32 (11.4%)<br><br>**Household income (USD) n (%):** Less than $5000: 57 (20.3%); $5001 to $15 000: 28 (10.0%); $15 001 to $30 000: 54 (19.2%); $30 001 to $50 000: 47 (16.7%); $50 001 to $75 000: 24 (8.5%); $75 001 to $90 000: 17 (5.7%); $90 001 to $119 999: 24 (8.5%); More than $120 000: 27 (9.6%); Not reported: 3 (1.1%)<br><br>**Caregiver employment Status n (%):** Employed: 181 (64.4%); Full time: 127 (45.2%); Part time: 54 (19.2%)
Romanelli 2023 [4]	United Kingdom	585 caretakers	No caregiver specific demographic details reported
Zhang 2022 [5]	China	Children with ILI symptoms: 1,913<br>6-11mo: 559<br>12-23mo: 735<br>24-35mo: 272<br>36-47mo: 203<br>48-59mo: 144	No caregiver specific demographic details reported
Lai 2021 [6]	China	Caregivers of patients with ILI:<br>Children aged 6–59 months: 3,049;<br>Chronic disease patients aged 18–59 years: 291;<br>Elderly aged 60+ years: 489;	**Household monthly per capita income (CNY 1000), Mean (SD):** 2.66 (2.76)
Wang 2021 [7]	China	Children in:<br>Outpatient: 799<br>Inpatient: 436	No caregiver specific demographic details reported
Rao 2020 [8]	USA	411	No caregiver specific demographic details reported

Study Details		Study Cohort Population, N	Caregiver-specific details
Study, Year	Location		
Salcedo-Mejía 2019 [9]	Colombia	44	No caregiver specific demographic details reported
Willis 2019 [10]	Australia	Total: 1,191<br>Influenza-positive: 238<br>Influenza-negative: 953<br>Other respiratory virus-positive: 670	**No. of adults in household, mean ± SD:** All participant: 2.09 ± 0.63; Influenza-positive: 2.12 ± 0.68; Influenza-negative: 2.08 ± 0.62; Other respiratory virus-positive: 2.05 ± 0.62<br><br>**Smoker in household, n (%):** All participants: 205 (17.5%); Influenza-positive: 38 (16.5%); Influenza-negative: 167 (17.7%); Other respiratory virus-positive: 125 (18.8%)
Streng 2018 [11]	Germany	Children with influenza:<br>All patients: 217<br>A/H3N2: 122<br>A/H1N1: 56<br>Influenza B: 39<br>Mild influenza: 29<br>Moderate-to-severe: 188	**Family size median (IQR):** 4 (3-4)<br>**Both parents working n (%):** 149 (70.0)
Aykac 2017 [12]	Turkey	132<br>Influenza positive: 15<br>Influenza negative: 117	**Influenza Positive:**<br>**Work status of fathers, n (%):** On leave: 5 (33.3%); Not on leave: 9 (60%); Unemployed: 1 (6.6%)<br>**Work status of mothers, n (%):** On leave: None; Not on leave: None; Unemployed: 15 (100%)<br>**Influenza Negative:**<br>**Work status of fathers, n (%):** On leave: 39 (33.3%); Not on leave: 77 (65.8%); Unemployed: 1 (6.6%)<br>**Work status of mothers, n (%):** On leave: 17 (14.5%); Not on leave: 0; Unemployed: 100 (85.4%)
Thorrington 2017 [13]	United Kingdom	34 children with ILI symptoms	A total of 74 questionnaires (85.1%) were completed by 2-parent families and 13 (14.9%) by single parent families. Of the 161 adults identified as parents or guardians in the survey, 78 were in full-time employment (48.4%), 33 (20.5%) were in part-time, shift, or casual employment, 25 (15.5%) were self-employed, 18 (11.2%) were unemployed, 2 (1.2%) were retired, and 4 (2.5%) were students, with 1 missing response.
Fragaszy 2017 [14]	United Kingdom	All people: 2,919<br>All illnesses: 4,818<br>ILI: 2,013; 0-15yrs: 555; 16-64 yrs: 1,169; 65+yrs: 270<br>Individuals tested for Flu A&B: 3,161<br>Influenza A PCR+: 177; 0-15yrs: 68; 16-64yrs: 99; 65+yrs: 8<br>Influenza B PCR+: 45; 0-15 yrs: 26; 16-64yrs: 15; 65+yrs: 4	No caregiver specific demographic details reported

Study Details		Study Cohort Population, N	Caregiver-specific details
Study, Year	Location		
Heikkinen 2016 [15]	Finland	Parents of children with:<br>Moderate-to-severe influenza: 177<br><3 yrs: 77<br>3-13 yrs: 100<br><br>Mild influenza: 181<br><3 yrs: 40<br>3-13 yrs: 141	No caregiver specific demographic details reported
Mughini-Gras 2016 [16]	The Netherlands	Children with ILI: 1,887<br>Children without ILI (n = 6842)	In total, 7268 mothers [median age 34 years, interquartile range (IQR), 31–37] and 1500 fathers (37 years; IQR, 33–41) were enrolled in the study.<br><br>25.54% and 19.82% of children with IL I had Having a parent working in health care and childcare compared to 22.22% and 17.19% in children without ILI
Tinoco 2015 [17]	Peru	Total cases of influenza: 1,321<br>Based on level of care:<br>Self care: 345<br>Seek non-medical attention: 351<br>Outpatients: 575<br>Emergency ward: 39<br>Hospitalized: 11	**Monthly household income (US$) n (%):** <$300: 296 (22%); $300–<$500: 264 (20%); $500–<$1000: 34(26%); ≥$1000: 202 (15%); Refused, unknown, missing: 213 (16%)
Silvennoinen 2015 [18]	Finland	Total children with influenza: 358<br>A/H1N1: 203<br>A/H3N2: 96<br>B: 95	No caregiver specific demographic details reported
Enserink 2014 [19]	The Netherlands	Day care center (DCC_-attending children with ILI: 417<br><br>Non DCC-attending children with ILI: 359	**DCC group (n=1930)**<br>Gender, male: 18.0%<br>Both parents employed: 94.5%<br>Working hours/month, mean: 26.5 hours<br>Household income >Euro3600 month: 39.3%<br>University diploma in household: 73.4%<br><br>**Non-DCC group (n=1997)**

Study Details		Study Cohort Population, N	Caregiver-specific details
**Study, Year**	**Location**		
			Gender, male: 18.7%<br>Both parents employed: 73.9%<br>Working hours/month, mean: 18.8 hours<br>Household income >Euro3600 month: 15.4%<br>University diploma in household: 51.6%
Silva 2014 [20]	France	201	No caregiver specific demographic details reported
Bhuiyan 2014 [21]	Bangladesh	Total patients with ILI: 173<br>Hospitalized: 41<br>Outpatients: 132	**Household monthly income (US$), median (IQR):** Outpatients: 129 (86–176); Hospitalized patients: 136 (100–186)<br>**Number of household members, median (IQR):** Outpatients: 5 (4-6); Hospitalized patients: 5 (4-7)
Wang 2013 [22]	China	Total (N): 1,537<br>Medical setting<br>Outpatient clinics: 1,005<br>ED: 532<br><br>Influenza Virus<br>Positive: 365<br>Negative: 1,172	**Total family income prior year (thousand U.S Dollars) n (%):** <$15.9k: 668 (57.7%); $15.9k-23.8k: 287 (24.8%); $23.8k-$31.7k: 118 (10.2%); $31.7k-39.7: 39 (3.4%); >$39.7k: 46 (4.0%)
Yin 2013 [23]	Australia	124 ILIs in 105 children (13 had 2 ILIs, 3 children had 3 ILIs)	**Proportion of mothers of study children living with a partner (married or de facto):** 97%<br>**Parents employment, % (*n*):** Both working: 73% (262); One working: 26% (92); Neither working: 1% (3)<br>**Parents' education [two-parent households% (n)] (348 households):** Both university: 51% (174); Both other: 20% (68); 1 university, 1 other: 29% (98)<br>**Parents education [single-parent households% (n)] (10 households):** university: 40% (4); other: 60% (6)<br>**Household income >$2000 per week:** 75 (264/351)
Ambrose & Antonova 2013 [24]	Study 1: Belgium, Finland, Israel, Spain, UK<br>Study 2: Belgium, the Czech Republic, Finland, Germany, Israel, Italy, Poland, Spain, Switzerland, UK<br><br>Study 3: Belgium, Finland, Germany, Greece, Israel, Italy, the	Subjects with confirmed influenza [total enrolled] in studies<br><br>Study 1:<br>LAIV—11 [490] (year 1), 21 [570] (year 2);<br>Placebo — 55 [356] (year 1), 123 [403] (year 2)<br><br>Study 2:	No caregiver specific demographic details reported

Study Details		Study Cohort Population, N	Caregiver-specific details
Study, Year	Location		
	Netherlands, Norway, Poland, Portugal, Spain, Switzerland, UK	LAIV— 23 [790];<br>IIV — 46 [818]<br><br>Study 3:<br>LAIV— 50 [1,114];<br>IIV — 73 [1,115]	
Galante 2012 [25]	Spain	Patients with influenza A:<br>Inpatients: 172<br>Outpatients: 224	No caregiver specific demographic details reported
Chiu 2012 [26]	China	Influenza A: 102<br>Influenza B: 45	No caregiver specific demographic details reported
Ortega-Sanchez 2012 [27]	USA	Children with influenza: N<br>Total: 281<br>Inpatients: 67<br>ED-patients: 122<br>Outpatients: 92	**Household income n (%):**<br>**Inpatients:** <$10,000: 25 (37%); $10,000 to <$30,000: 13 (19%); $30,000 to <$50,000: 7 (10%); $50,000 to <$100,000: 9 (13%); ≥$100,000: 4 (6%); Refused/Unknown/Missing: 11 (16%)<br>**ED-patients**: <$10,000: 29 (24%); $10,000 to <$30,000: 37 (30%); $30,000 to <$50,000: 9 (8%); $50,000 to <$100,000: 8 (7%); ≥$100,000: 8 (7%); Refused/Unknown/Missing: 35 (29%)<br>**Outpatients:** <$10,000: 29 (32%); $10,000 to <$30,000: 18 (20%); $30,000 to <$50,000: 8 (9%); $50,000 to <$100,000: 11(12%); ≥$100,000: 5 (5%); Refused/Unknown/Missing: 27 (29%)
Esposito 2011a [28]	Italy	Child's diagnosis cases by aetiology and age: N<br><br>Influenza-negative: 4,845<br>Influenza-positive: 2,143<br>Influenza-positive <2yrs: 343<br>Influenza-positive 2-5yrs: 1,071<br>Influenza-positive >5yrs: 729<br><br>Influenza-positive patients by viral type:<br>Influenza A-positive: 1,751<br>Influenza B-positive: 392	No caregiver specific demographic details reported

Study Details		Study Cohort Population, N	Caregiver-specific details
Study, Year	Location		
Esposito 2011b [29]	Italy	Households of seasonal influenza-positive children (n)<br>A/H1N1, Season 2007/2008: 325<br>A/H3N2, Season 2008/2009: 1,217<br>A/H1N1/2009, Season 2009/2010: 1,005	No caregiver specific demographic details reported
Heinonen 2010 [30]	Finland	Total: 98<br>Any influenza: 98<br>Oseltamivir: 37<br>Placebo: 61<br><br>Any influenza in unvaccinated: 85<br>Oseltamivir: 34<br>Placebo: 51<br><br>Influenza A: 79<br>Oseltamivir: 29<br>Placebo: 50<br><br>Influenza A in unvaccinated: 67<br>Oseltamivir: 26<br>Placebo: 41<br><br>Influenza B: 19<br>Oseltamivir: 8<br>Placebo: 11	No caregiver specific demographic details reported
Palmer 2010 [31]	USA	Child household with ILI = 1,232/3,814 of total sample<br>Household with ILI N = 800/2,233 of total sample	mean household size: 4<br>Avg age of employee and other adult household members: 41.7 (21-64) and 39.9 (19-93)<br>Total household income, mode: $50,000–$74,999<br>Among employees, 86% were white, 31% female, and 41% had attained a college degree

Study Details		Study Cohort Population, N	Caregiver-specific details
Study, Year	Location		
Iskander 2009 [32]	Australia	273 total sample size, wherein parents of 260 children completed survey	No caregiver specific demographic details reported
Bourgeois 2009 [33]	USA	Children with<br>Influenza: 59<br>RSV: 151	No caregiver specific demographic details reported
Johnson 2008 [34]	USA	315	**Households (n=220), n (%):** Single adult home: 37 (17%), Two-adult home: 145 (66%), Three- or four- adult home: 38 (17)<br>**All adults employed outside the home, n (%):** 118 (54%)<br>**Occupations of those employed outside home, n (%):** Healthcare: 35 (11%); Education: 36 (11%); Industry: 27 (9%)<br>**Number of homes where all adults in home employed, n (%):** 118 (54%)
Li 2007 [35]	USA	12850 households	<u>**Not experiencing influenza**</u> (characteristics of households)<br><br>**More family members (>4), (%±se):** 25.78±0.62<br>**More adults (>2), (%±se):** 15.85±0.52<br>**More workers (>2), (%±se):** 16.34±0.51<br>**Below poverty-line, (%±se):** 5.14±0.32<br>**Medically insured, (%±se):** 82.09±0.62<br>**Metropolitan areas, (%±se):** 81.10±1.05<br>**None retired, (%±se):** 98.64±0.13<br>**Single parent, (%±se):** 24.30±0.63<br>**Unemployed, (%±se):** 27.98±0.70<br><br><u>**Experiencing influenza**</u><br>**More family members (>4), (%±se):** 29.66±1.32<br>**More adults (>2), (%±se):** 14.54±1.05<br>**More workers (>2), (%±se):** 16.16±1.11<br>**Below poverty-line, (%±se):** 4.09±0.52<br>**Medically insured, (%±se):** 84.54±0.98<br>**Metropolitan areas, (%±se):** 77.64±1.87<br>**None retired, (%±se):** 98.93±0.28<br>**Single parent, (%±se):** 18.31±1.18<br>**Unemployed, (%±se):** 25.15±1.42
Ploin 2007 [36]	France	Total children: 575<br>With positive influenza detection: 283 (follow-up 263)	No caregiver specific demographic details reported

Study Details		Study Cohort Population, N	Caregiver-specific details
Study, Year	Location		
		With negative influenza detection: 292 (follow-up 275)	

Table S9. Results, Work Productivity.

Study Details		Study Cohort Population, N	Results
Study, Year	Location		Work Productivity Outcomes
Overmann 2023 [3]	USA	281 caregivers	**Caregiver missed work: N (%)**<br>Total: 129 (45.9)<br><br>**Workdays missed**<br>1: 49 (17.4)<br>2: 37 (13.2)<br>≥3: 38 (13.5)<br>Unspecified: 5 (1.8)<br><br>**Caregiver time caring for child**<br>The same: 34 (12.1)<br>Slightly more than usual: 87 (31.0)<br>Much more than usual: 160 (56.9)
Romanelli 2023 [4]	United Kingdom	585 caretakers	Among 585 survey respondents who reported caring for a dependent with influenza while employed, 355 (61%) took at least some time from work to care for them, averaging 2.0 days off work (SE ±1.7 days); Median = 1.4 days (IQR = 1–3 days).<br><br>Older working adults were less likely to take time off from work than younger adults (44.9% aged 50–64 years vs 63.5% aged 18–49 years). However, the time they took off work was longer, averaging 2.3 days (SE ±1.8) vs 2.0 (SE ±1.7), respectively. Median = 2 days (IQR = 0.9–3 days) and 1.3 days (IQR = 0.9–2.6 days) for those aged 50–64 and 18–49, respectively.
Zhang 2022 [5]	China	Children with ILI symptoms: 1,913<br>6-11mo: 559<br>12-23mo: 735<br>24-35mo: 272<br>36-47mo: 203<br>48-59mo: 144	1,030 (57.3%) of family members asked for leave, absenteeism for a median (IQR) of 3 (2, 5) days per sick child<br><br>**Absenteeism N (%) and median (IQR) days absenteeism by patient's age group in months:**<br>6–11 mo: 274 (52.2%), 2 (1, 2)<br>12–23 mo: 383 (55.3%), 4.5 (2.5, 7)<br>24–35 mo: 150 (58.8%), 3 (2, 7)<br>36–47 mo: 121 (64.4%), 5 (2, 7.5)<br>48–59 mo: 102 (75.0%), 5 (3, 10)

Study Details		Study Cohort Population, N	Results
**Study, Year**	**Location**		**Work Productivity Outcomes**
Lai 2021 [6]	China	Caregivers of patients with ILI:<br>Children aged 6–59 months: 3,049;<br>Chronic disease patients aged 18–59 years: 291;<br>Elderly aged 60+ years: 489;	**Lost labor days of respondents' families: mean (95% CI)**<br>Children aged 6–59 months: 2 (1.9-2.1)<br>Chronic disease patients aged 18–59 years: 0.7 (0.4-1.0)<br>Elderly aged 60+ years: 2 (1.5, 2.6)
Wang 2021 [7]	China	Children in:<br>Outpatient: 799<br>Inpatient: 436	**Median (IQR) days of productivity loss for caregivers:**<br><br>Outpatient, 0 (0-2)<br>Inpatient, 7 (6-9)
Rao 2020 [8]	USA	411	209 parents of total 411 children reported absenteeism<br>Parent absenteeism days Median (IQR): 1.0 day (0.0-3.0)
Salcedo-Mejía 2019 [9]	Colombia	44	Indirect costs were reported by all 17 interviewed caregivers. Most were mothers (70.6%) and housewives (83.3%), and paid work was reported by 17%. All fathers (23.5%) were engaged in paid work.<br><br>The average number of days caring for hospitalized children was 10.2 days (95% CI, 5.4-14.9) in mothers and 1.5 days (95% CI, 0.6-2.4) in fathers.<br><br>The average indirect cost was $118, and the median total indirect cost was $82.10 (IQR $41.10-$133.40
Willis 2019 [10]	Australia	Total: 1,191<br>Influenza-positive: 238<br>Influenza-negative: 953<br>Other respiratory virus-positive: 670	**Parents with work absenteeism; mean (SD) hours:**<br>Influenza-positive, 109 (53.4%); 28.5 (27.8), calculated as 3.5<br>Influenza-negative, 449 (55.2%); 23.0 (23.9), calculated as 2.9 workdays<br>Other respiratory virus-positive, 329 (57.6%); 22.7 (24.1), calculated as 2.8 workdays<br><br>Parents of children with influenza missed 8.24 hours more work (95% CI 1.85-14.62) than parents of children with another respiratory virus (P = 0.012).
Streng 2018 [11]	Germany	Children with influenza:<br>All patients: 217<br>A/H3N2: 122<br>A/H1N1: 56<br>Influenza B: 39	**Parent workdays lost after practice visit, median (IQR)**<br>All patients: 4 (2-6)<br>A/H3N2: 4 (2.5-5)<br>A/H1N1: 3 (2-6)<br>Influenza B: 4 (2.5-6)

Study Details		Study Cohort Population, N	Results
Study, Year	Location		Work Productivity Outcomes
		Mild influenza: 29<br>Moderate-to-severe: 188	Mild influenza: 3 (2-5) days<br>Moderate-to-severe: 4 (3-6) days
Aykac 2017 [12]	Turkey	132<br>Influenza positive: 15<br>Influenza negative: 117	Influenza-positive patients:<br>Fathers on leave, 5 (36%)<br>Mothers on leave, none were employed<br><br>Influenza-negative patients:<br>Fathers on leave, 39 (34%)<br>Mothers on leave, all 17 on leave (100%) + 100<br><br>10 mothers (71.4%) and nine fathers (60%) experienced sleep disturbances at night, and both the mothers and fathers of six patients (40%) experienced sleep disturbances at night.
Thorrington 2017 [13]	United Kingdom	34 children with ILI symptoms	**Mean days of absenteeism for caregivers of children with ILI symptoms:**<br>3.7 (95% CI: 2.7–4.8)<br><br>Children with symptoms consistent with ILI were absent from school for a mean duration of 3.8 days (95% CI: 3.0-4.8). During this time, they were looked after by a mean of 1.7 caregivers (95% CI: 1.4-2.0)
Fragaszy 2017 [14]	United Kingdom	All people: 2,919<br>All illnesses: 4,818<br>ILI: 2,013; 0-15yrs: 555; 16-64 yrs: 1,169; 65+yrs: 270<br>Individuals tested for Flu A&B: 3,161<br>Influenza A PCR+: 177; 0-15yrs: 68; 16-64yrs: 99; 65+yrs: 8<br>Influenza B PCR+: 45; 0-15 yrs: 26; 16-64yrs: 15; 65+yrs: 4	**% of illnesses where someone else takes time off to care for ill participants: N (%) and mean (min, max) days**<br>**Overall**<br>ILI: 11%, 2.0 (1,7) days; Flu A+: 28%, 2.7 (1,6) days; Flu B+: 29%, 1.6 (1,2) days<br>**Children (0-15y)**<br>ILI:24%, 2.2 (1,7) days; Flu A+: 70%, 2.9 (1,6) days; Flu B+: 42%, 1.6 (1,2) days<br>**Adults (16-64y)**<br>ILI: 7%, 1.5 (1,5) days; Flu A+: 10%, 2.0 (1,3) days; Flu B+: 0%, 0 (NA)<br>**Older adults (65+ y):**<br>ILI:6%, 2.5 (1,5) days; Flu A+: 0%, 0; Flu B PCR+: 0, 0
Heikkinen 2016 [15]	Finland	Parents of children with:<br>Moderate-to-severe influenza: 177<br><3 yrs: 77	**Parents who missed ≥1 workday: N (%), mean (SD) days**<br>**Moderate-to-severe:** all children: 103/177 (58%), 3.2 (1.8) days ; <3 years: 49/77 (64%), 3.7 (2.0) ; 3-13 years: 54/100 (54%), 2.7 (1.5)<br>**Mild:** all children: 88/181 (49%), 2.8 (1.5); <3 years: 28/40 (70%), 3.1 (1.6); 3-13 years: 60/141 (43%), 2.6 (1.5)

Study Details		Study Cohort Population, N	Results
Study, Year	Location		Work Productivity Outcomes
		3-13 yrs: 100<br><br>Mild influenza: 181<br><3 yrs: 40<br>3-13 yrs: 141	**Total missed days per 100 children (95% CI)**<br>**Moderate-to-severe:** all children: 184 (153-215); <3 years: 236 (182-290); 3-13 years: 144 (110-178)<br>**Mild:** all children: 135 (109-161); <3 years: 218 (154-281); 3-13 years: 111 (84-138)
Mughini-Gras 2016 [16]	The Netherlands	Children with ILI: 1,887	Absenteeism; N (%) median (IQR) days:<br>Child's illness, 309 (16%); 1 (1-2)
Tinoco 2015 [17]	Peru	Total cases of influenza: 1,321<br>Based on level of care:<br>Self care: 345<br>Seek non-medical attention: 351<br>Outpatients: 575<br>Emergency ward: 39<br>Hospitalized: 11	**Total cases of influenza median (IQR) days:** work place - 2.3 (2.5); Unpaid activity - 1 (1)<br><br>**Based on level of care:**<br>Self care: work place - 1.5 (1.5); Unpaid activity - 0.8 (0.75)<br>Seek non-medical attention: work place - 2.5 (2.3); Unpaid activity - 0.8 (0.8)<br>Outpatients: work place - 2.3 (1.5); Unpaid activity - 1 (1.25)<br>Emergency ward: work place - 5 (4.6); Unpaid activity - 1 (0.3)<br>Hospitalized: work place - 2.8 (NR); Unpaid activity - 3.8 (4.8)
Silvennoinen 2015 [18]	Finland	Total children with influenza: 358<br>A/H1N1: 203<br>A/H3N2: 96<br>B: 95	147 (49%) of children with influenza had ≥1 parent stay off work for ≥1 day due to child's influenza<br>412 total days of absenteeism<br>138 days per 100 children with influenza (95% CI: 117–159)<br><br>Mean (SD) workdays missed, all children:<br>Influenza A/H1N1, 2.8 (0.2)<br>Influenza A/H3N2, 2.8 (0.2)<br>Influenza B, 2.7 (0.2)
Enserink 2014 [19]	The Netherlands	Day care center (DCC_-attending children with ILI: 417<br><br>Non DCC-attending children with ILI: 359	Both households with and without children attending a DCC experienced approximately 1.5 days of work days lost per episode of GE and ILI if productivity losses were involved.
Silva 2014 [20]	France	201	One in four parents (children < 14 years) were out of work for approximately 2.8 days
Bhuiyan 2014 [21]	Bangladesh	Total patients with ILI: 173<br>Hospitalized: 41<br>Outpatients: 132	Hospitalized: 38 caregivers, including 34 homemakers, missed a median of seven productive days (IQR = 4–12) at a median of US$1·42 (IQR = 1·42–1·42) per day.<br><br>Outpatients: Homemakers were the primary caregivers for ambulatory case-patients, but all reported carrying out their usual activities without interruption or with modest disruption while caring for ill family members, so no

Study Details		Study Cohort Population, N	Results
Study, Year	Location		Work Productivity Outcomes
			value was attributed to these events. Other caregivers such as fathers, husbands, and grandparents (n = 7) missed a median of one workday (IQR = 1–3) at a median cost of US$1·42 (IQR = 0·47–2·14) per day.
Wang 2013 [22]	China	Total (N): 1,537<br>Medical setting<br>Outpatient clinics: 1,005<br>ED: 532<br><br>Influenza Virus<br>Positive: 365<br>Negative: 1,172	Parent or guardian of 1,341 ILI cases (86.7%) completed follow-up survey; the remaining 196 were either lost to follow-up or the patient's family member refused to participate in the additional survey. In total, 1,333 ILI cases were included in the non-medical costs and indirect costs analysis.<br><br>**Mean (SD) missed workdays for parents:**<br>Influenza-positive, 1.8 (2.6)<br>Influenza-negative, 1.7 (2.4)
Yin 2013 [23]	Australia	124 ILIs in 105 children (13 had 2 ILIs, 3 children had 3 ILIs)	Time off work for caregivers per ILI episode: Mean 12·9 hours (median 4 hours). Of the mean ILI cost, AU$406 (65%; €227, US$268 or £179) was due to carer time off work.<br><br>ILI-related time away from recreation for caregivers: Mean 3·1 hours (median 0 hour)
Ambrose & Antonova 2013 [24]	Study 1: Belgium, Finland, Israel, Spain, UK<br>Study 2: Belgium, the Czech Republic, Finland, Germany, Israel, Italy, Poland, Spain, Switzerland, UK<br><br>Study 3: Belgium, Finland, Germany, Greece, Israel, Italy, the Netherlands, Norway, Poland, Portugal, Spain, Switzerland, UK	Subjects with confirmed influenza [total enrolled] in studies<br><br>Study 1:<br>LAIV —11 [490] (year 1), 21 [570] (year 2);<br>Placebo — 55 [356] (year 1), 123 [403] (year 2)<br><br>Study 2:<br>LAIV — 23 [790];<br>IIV —46 [818]<br><br>Study 3:<br>LAIV — 50 [1,114];<br>IIV —73 [1,115]	**Any missed parental working days % of subjects with confirmed influenza, mean missed work days**<br><br>Study 1:<br>LAIV: Year 1 - 55% of 11, 1.8 days; Year 2 - 29% of 21, 2.3 days<br>Placebo: Year 1 - 51% of 55, 2.8 days; Year - 44% of 123, 2.7 days<br><br>Study 2:<br>LAIV: 57% of 23, 3.0 days<br>IIV: 83% of 46; 4.3 days<br><br>Study 3:<br>LAIV: 82% of 50, 3.8 days<br>IIV: 88% of 73, 3.7 days
Galante 2012 [25]	Spain	Patients with influenza A:<br>Inpatients: 172<br>Outpatients: 224	**Patients whose caregivers required work absenteeism: N(%); Mean (SD) days:**<br>Inpatients: 38/172 (21.7%); 10.7 (14.1)<br>Outpatients: 19/224 (8.5%); 4.1 (4.1)
Chiu 2012 [26]	China	Influenza A: 102<br>Influenza B: 45	**Children who had a parent miss work due to child's illness: N (%)**<br>Influenza A, 36 (35%)

Study Details		Study Cohort Population, N	Results
Study, Year	Location		Work Productivity Outcomes
			Influenza B, 20 (44%)<br><br>**Mean (SD) workdays missed:**<br>Influenza A, 2.43 (1.53)<br>Influenza B, 2.26 (1.27)
Ortega-Sanchez 2012 [27]	USA	Children with influenza: N<br>Total: 281<br>Inpatients: 67<br>ED-patients: 122<br>Outpatients: 92	**Caregivers reporting time off of work: N (%); total mean (SD) hours**<br>Inpatients: 50 (75%); 73 (68), calculated as 9.1 workdays; median (IQR): 55 (66) hrs<br>ED patients: 57 (47%); 19 (42), calculated as 2.4 workdays; median (IQR): 6 (13) hrs<br>Outpatients, 39 (42%); 11 (17); calculated as 1.4 workdays; median (IQR): 0 (15) hrs
Esposito 2011a [28]	Italy	Child's diagnosis cases by aetiology and age: N<br><br>Influenza-negative: 4,845<br>Influenza-positive: 2,143<br>Influenza-positive <2yrs: 343<br>Influenza-positive 2-5yrs: 1,071<br>Influenza-positive >5yrs: 729	**ILI and related morbidity among households in the 7 days following a child's diagnosis:**<br><br>**Mothers who remained absent from work, N (%); mean (SD) days:**<br>Influenza-negative: 579 (12.0%); 3.39 (2.26)<br>Influenza-positive: 349 (16.3%); 4.46 (2.11)<br>Influenza-positive <2yrs: 49 (14.3%); 4.95 (2.61)<br>Influenza-positive 2-5yrs: 185 (17.3%); 4.88 (2.03)<br>Influenza-positive >5yrs: 115 (15.8%); 1.91 (2.34)<br><br>**Fathers who remained absent from work, N (%); mean (SD) days:**<br>Influenza-negative: 96 (2.0%); 1.96 (2.04)<br>Influenza-positive: 130 (6.1%); 4.31 (2.73)<br>Influenza-positive <2yrs: 19 (5.5%); 5.61 (2.64)<br>Influenza-positive 2-5yrs: 74 (6.9%); 4.99 (2.88)<br>Influenza-positive >5yrs: 18 (2.5%); 1.98 (2.06)
		Influenza-positive patients by viral type:<br>Influenza A-positive: 1,751<br>Influenza B-positive: 392	**Impact on household by viral type:**<br><br>**Mothers who remained absent from work, N (%); mean (SD) days:**<br>Influenza A-positive: 316 (18.0%); 4.57 (2.43)<br>Influenza B-positive: 33 (8.4%); 2.99 (1.90)<br><br>**Fathers who remained absent from work, N (%); mean (SD) days:**<br>Influenza A-positive: 122 (7.0%); 4.41 (3.10)<br>Influenza B-positive: 8 (2.0%); 3.00 (2.71)

Study Details		Study Cohort Population, N	Results
**Study, Year**	**Location**		**Work Productivity Outcomes**
Esposito 2011b [29]	Italy	Households of seasonal influenza-positive children (n)<br>A/H1N1, Season 2007/2008: 325<br>A/H3N2, Season 2008/2009: 1,217<br>A/H1N1/2009, Season 2009/2010: 1,005	**Households of seasonal influenza-positive children participated in survey**<br>A/H1N1, Season 2007/2008: 321 (98.8%)<br>A/H3N2, Season 2008/2009: 1,205 (99.0%)<br>A/H1N1/2009, Season 2009/2010: 995 (99.0%)<br><br>**Working days lost by mothers, mean (SD)**<br>A/H1N1, Season 2007/2008: 3.9 (2.1)<br>A/H3N2, Season 2008/2009: 5.9 (2.6)<br>A/H1N1/2009, Season 2009/2010: 5.9 (2.6)<br><br>**Working days lost by fathers, mean (SD)**<br>A/H1N1, Season 2007/2008: 1.2 (1.4)<br>A/H3N2, Season 2008/2009: 3.4 (2.1)<br>A/H1N1/2009, Season 2009/2010: 3.3 (2.6)
Heinonen 2010 [30]	Finland	Total: 98<br>Any influenza: 98<br>Oseltamivir: 37<br>Placebo: 61<br><br>Any influenza in unvaccinated: 85<br>Oseltamivir: 34<br>Placebo: 51<br><br>Influenza A: 79<br>Oseltamivir: 29<br>Placebo: 50<br><br>Influenza A in unvaccinated: 67<br>Oseltamivir: 26<br>Placebo: 41	Parental absence from work, median days (IQR)<br>Any influenza<br>Oseltamivir: 0.0 (0.0-2.0)<br>Placebo: 2.0 (0.0-4.0); difference days 2.0, p=0.01<br><br>Any influenza in unvaccinated:<br>Oseltamivir: 0.0 (0.0-2.0)<br>Placebo: 2.0 (0.0-4.0); difference days 2.0, p=0.02<br><br>Influenza A<br>Oseltamivir: 0.0 (0.0-2.0)<br>Placebo: 3.0 (0.0-4.0); difference days 3.0, p=0.007<br><br>Influenza A in unvaccinated:<br>Oseltamivir: 0.0 (0.0-2.0)<br>Placebo: 3.0 (0.0-4.0); difference days 3.0, p=0.01<br><br>Influenza B:

Study Details		Study Cohort Population, N	Results
Study, Year	Location		Work Productivity Outcomes
		Influenza B: 19<br>Oseltamivir: 8<br>Placebo: 11	Oseltamivir: 1.0 (0.0-3.0)<br>Placebo: 1.0 (0.0-3.0); difference days 0.0, p=0.97
Palmer 2010 [31]	USA	Child household with ILI = 1,232/3,814 of total sample<br>Household with ILI N = 800/2,233 of total sample	**Missed ≥1 workday; mean days due to ILI:**<br>Household ILI, 30%; 0.5<br>Child household ILI, 31%; 0.5<br><br>**Mean (SD) hours ILI-related presenteeism:**<br>Household ILI, 1.3 (3.5)<br>Child household ILI, 1.4 (3.7)
Iskander 2009 [32]	Australia	273 total sample size, wherein parents of 260 children completed survey	71% of influenza cases had ≥1 parent who needed time off work to look after a sick child<br>Mean days off work, 3.2<br>Median days off work, 2.0
Bourgeois 2009 [33]	USA	Children with<br>Influenza: 59<br>RSV: 151	Almost two thirds of caregivers missed at least 1 day of work as a result of the child's illness. The estimated total number of workdays missed per year on a national basis by caregivers of children with influenza was 246,965 days.<br><br>Proportion (95% CI) Among Prospective Patients: 64 (53–77)<br><br>Yearly Rate (95% CI) in Massachusetts Population/1000 Children: 5.5 (2.0–14.7)<br><br>Yearly Rate (95% CI) in US Population/1000 Children: 6.5 (2.5–17.2)
Johnson 2008 [34]	USA	315	Missed ≥1 workday; median (range) days:<br>Overall, 76/315 (24%); 3.0 (1-14)<br>Own illness, 36 (47%)<br>As caregiver, 18 (24%)
Li 2007 [35]	USA	12850 households	Overall: ILI households lost 1.12 more workdays (95% CI: 0.20–2.04) vs non-ILI households<br>Among fully employed and insured households: ILI lost an additional 0.89 (0.48–1.30) for ILI vs no-ILI households
Ploin 2007 [36]	France	Total children: 575<br>With positive influenza detection: 283 (follow-up 263)<br>With negative influenza detection: 292 (follow-up 275)	**Parental absenteeism: N (%) and mean (SD) days**<br><br>Influenza positive: 142 (54%), 6.3 (4.7)<br><br>Influenza negative: 149 (54%), 5.9 (4.3)

References

1. Chow, M.Y.; Morrow, A.; Heron, L.; et al. Quality of life for parents of children with influenza-like illness: Development validation of, Care-ILI-QoL. *Qual. Life Res.* **2014**, *23*, 939–951.
2. Chow, M.Y.; Yin, J.K.; Heron, L.; Heron, L.; Morrow, A.; Dierig, A.; Booy, R.; Leask, J. The impact of influenza-like illness in young children on their parents: A quality of life survey. *Qual. Life Res.* **2014**, *23*, 1651–1660.
3. Overmann, K.M.; Porter, S.C.; Zhang, Y.; Britto, M.T. Caregiver Quality of Life During Pediatric Influenza-Like Illness: A Cross-Sectional Study During the COVID-19 Pandemic. *J. Patient Exp.* **2023**, *10*, 23743735231188840.
4. Romanelli, R.J.; Cabling, M.; Marciniak-Nuqui, Z.; Marjanovic, S.; Morris, S.; Dufresne, E.; Yerushalmi, E. The Societal and Indirect Economic Burden of Seasonalinfluenza in the United Kingdom; RAND Corporation: Santa Monica, CA, USA, 2023.
5. Zhang, H.; Ren, X.; Tian, K.; Yu, J.; Zhu, A.; Zhang, L.; Gao, G.F.; Li, Z. The Impact and Vaccination Coverage of Seasonal Influenza among ChildrenAged 6–59 Months in China in 2017–2018: An Internet Panel Survey. *Vaccines* **2022**, *10*, 630.
6. Lai, X.; Rong, H.; Ma, X.; Hou, Z.; Li, S.; Jing, R.; Zhang, H.; Lyu, Y.; Wang, J.; Feng, H.; et al. The Economic Burden of Influenza-Like Illness among Children, Chronic DiseasePatients, and the Elderly in China: A National Cross-Sectional Survey. *Int. J. Environ. Res. Public Health* **2021**, *18*, 6277.
7. Wang, Y.; Chen, L.; Cheng, F.; Biggerstaff, M.; Situ, S.; Zhou, S.; Gao, J.; Liu, C.; Zhang, J.; Millman, A.J.; et al. Economic burden of influenza illness among children under 5 years inSuzhou, China: Report from the cost surveys during 2011/12 to 2016/17 influenza seasons. *Vaccine* **2021**, *39*, 1303–1309.
8. Rao, S.; Yanni, E.; Moss, A.; Lamb, M.M.; Schuind, A.; Bekkat-Berkani, R.; Innis, B.L.; Cotter, J.; Mistry, R.D.; Asturias, E.J. Evaluation of a New Clinical Endpoint for Moderate to Severe InfluenzaDisease in Children: A Prospective Cohort Study. *J. Pediatr. Infect. Dis. Soc.* **2020**, *9*, 460–467.
9. Salcedo-Mejía, F.; Alvis-Zakzuk, N.J.; Carrasquilla-Sotomayor, M.; Redondo, H.P.; Castañeda-Orjuela, C.; De la Hoz-Restrepo, F.; Alvis-Guzmán, N. Economic Cost of Severe Acute Respiratory Infection Associated to Influenza in Colombian Children: A Single Setting Analysis. *Value Health Reg. Issues* **2019**, *20*, 159–163.
10. Willis, G.A.; Preen, D.B.; Richmond, P.C.; acoby, P.; Effler, P.V.; Smith, D.W.; Robins, C.; Borland, M.L.; Levy, A.; Keil, A.D.; et al. The impact of influenza infection on young children, their familyand the health care system. *Influenza Other Respir. Viruses* **2019**, *13*, 18–27.
11. Streng, A.; Prifert, C.; Weissbrich, B.; Sauerbrei, A.; Schmidt-Ott, R.; Liese, J.G. Subtype-specific Clinical Presentation, Medical Treatment and Family Impact of Influenza in Children 1–5 Years of Age Treated in Outpatient Practices in Germany DuringThree Postpandemic Years, 2013–2015. *Pediatr. Infect. Dis. J.* **2018**, *37*, 861–867.
12. Aykaç, K.; Tanır Basaranoglu, S.; Gözmen, O.; Yetimakman, A.; Tekşam, Ö.; Kara, A. The Comfort Burden of Seasonal Influenza and Influenza-like Disease in Hospitalized Children: 2015–2016 Season. *J. Pediatr. Infect.* **2017**, *11*, 100–105.
13. Thorrington, D.; Balasegaram, S.; Cleary, P.; Hay, C.; Eames, K. Social and Economic Impacts of School InfluenzaOutbreaks in England: Survey of Caregivers. *J. Sch. Health* **2017**, *87*, 209–216.
14. Fragaszy, E.B.; Warren-Gash, C.; White, P.J.; Zambon, M.; Edmunds, W.J.; Nguyen-Van-Tam, J.S.; Hayward, A.C.; Flu Watch Group. Effects of seasonal and pandemic influenza on health-related quality of life, work and school absence in England: Results from the Flu Watch cohort study. *Influenza Other Respir. Viruses* **2018**, *12*, 171–182.
15. Heikkinen, T.; Silvennoinen, H.; Heinonen, S.; Vuorinen, T. Clinical and socioeconomic impact of moderate-to-severe versus mild influenza in children. *Eur. J. Clin. Microbiol. Infect. Dis.* **2016**, *35*, 1107–1113.
16. Mughini-Gras, L.; Pijnacker, R.; Enserink, R.; Heusinkveld, M.; van der Hoek, W.; van Pelt, W. Influenza-like Illness in Households with Children ofPreschool Age. *Pediatr. Infect. Dis. J.* **2016**, *35*, 242–248.
17. Tinoco, Y.O.; Azziz-Baumgartner, E.; Rázuri, H.; Ortiz, E.; Gomez, J.; Widdowson, M.A.; Uyeki, T.M.; Gilman, R.H.; Bausch, D.G. A population-based estimate of the economic burden ofinfluenza in Peru, 2009–2010. *Influenza Other Respir. Viruses* **2016**, *10*, 301–309.
18. Silvennoinen, H.; Huusko, T.; Vuorinen, T.; Heikkinen, T. Comparative Burden of Influenza A/H1N1, A/H3N2 and B Infections in Children Treated as Outpatients. *Pediatr. Infect. Dis. J.* **2015**, *34*, 1081–1085.
19. Enserink, R.; Lugnér, A.; Suijkerbuijk, A.; Bruijning-Verhagen, P.; Smit, H.A.; van Pelt, W. Gastrointestinal and Respiratory Illness in Children That Do andDo Not Attend Child Day Care Centers: A Cost-of-Illness Study. *PLoS ONE* **2014**, *9*, e104940.

20. Silva, M.L.; Perrier, L.; Späth, H.M.; Grog, I.; Mosnier, A.; Havet, N.; Cohen, J.M. Economic burden of seasonal influenza B in France during winter 2010–2011. *BMC Public Health* **2014**, *14*, 56.
21. Bhuiyan, M.U.; Luby, S.P.; Alamgir, N.I.; Sturm-Ramirez, K.; Gurley, E.S.; Zaman, R.U.; Alamgir, A.S.M. Economic burden of influenza-associated hospitalizations andoutpatient visits in Bangladesh during 2010. *Influenza Other Respir. Viruses* **2014**, *8*, 406–413.
22. Wang, D.; Zhang, T.; Wu, J.; Jiang, Y.; Ding, Y.; Hua, J.; Li, Y.; Zhang, J.; Chen, L.; Feng, Z.; et al. Socio-Economic Burden of Influenza among Children Younger than 5 Yearsin the Outpatient Setting in Suzhou, China. *PLoS ONE* **2013**, *8*, e69035.
23. Yin, J.K.; Salkeld, G.; Lambert, S.B.; Dierig, A.; Heron, L.; Leask, J.; Yui Kwan Chow, M.; Booy, R. Estimates and determinants of economic impacts from influenza-like illnesses caused by respiratory viruses in Australian children attending childcare: A cohort study. *Influenza Other Respir. Viruses* **2013**, *7*, 1103–1112.
24. Ambrose, C.S.; Antonova, E.N. The healthcare and societal burden associated with influenza in vaccinated andunvaccinated European and Israeli children. *Eur. J. Clin. Microbiol. Infect. Dis.* **2014**, *33*, 569–575.
25. Galante, M.; Garin, O.; Sicuri, E.; Cots, F.; García-Altés, A.; Ferrer, M.; Dominguez, À.; Alonso, J. Health Services Utilization, Work Absenteeism and Costs of Pandemic Influenza A (H1N1) 2009 in Spain: A Multicenter-Longitudinal Study. *PLoS ONE* **2012**, *7*, e31696.
26. Chiu, S.S.; Chan, K.-H.; So, L.Y.; Chen, R.; Chan, E.L.; Peiris, J.S.M. The population based socioeconomic burden of pediatric influenza-associated hospitalization in Hong Kong. *Vaccine* **2012**, *30*, 1895–1900.
27. Ortega-Sanchez, I.R.; Molinari, N.-A.M.; Fairbrother, G.; Szilagyi, P.G.; Edwards, K.M.; Griffin, M.R.; Cassedy, A.; Poehling, K.A.; Bridges, C.; Staat, M.A. Indirect, out-of-pocket and medical costs frominfluenza-related illness in young children. *Vaccine* **2012**, *30*, 4175–4181.
28. Esposito, S.; Cantarutti, L.; Molteni, C.G.; Daleno, C.; Scala, A.; Tagliabue, C.; Pelucchi, C.; Giaquinto, C.; Principi, N. Clinical manifestations and socio-economic impact of influenzaamong healthy children in the community. *J. Infect.* **2011**, *62*, 379–387.
29. Esposito, S.; Molteni, C.G.; Daleno, C.; Tagliabue, C.; Picciolli, I.; Scala, A.; Pelucchi, C.; Fossali, E.; Principi, N. Impact of pandemic A/H1N1/2009 influenza on children and their families: Comparison with seasonal A/H1N1 and A/H3N2 influenza viruses. *J. Infect.* **2011**, *63*, 300–307.
30. Heinonen, S.; Silvennoinen, H.; Lehtinen, P.; Vainionpää, R.; Vahlberg, T.; Ziegler, T.; Ikonen, N.; Puhakka, T.; Heikkinen, T. Early oseltamivir treatment of influenza in children 1–3 yearsof age: A randomized controlled trial. *Clin. Infect. Dis.* **2010**, *51*, 887–894.
31. Palmer, L.A.; Rousculp, M.D.; Johnston, S.S.; Mahadevia, P.J.; Nichol, K.L. Effect of influenza-like illness and otherwintertime respiratory illnesses on worker productivity: The child and household influenza-illness and employee function (CHIEF) study. *Vaccine* **2010**, *28*, 5049–5056.
32. Iskander, M.; Kesson, A.; Dwyer, D.; Rost, L.; Pym, M.; Wang, H.; McCaskill, M.; Booy, R. The burden of influenza in children under 5 years admitted to theChildren's Hospital at Westmead in the winter of 2006. *J. Paediatr. Child Health* **2009**, *45*, 698–703.
33. Bourgeois, F.T.; Valim, C.; McAdam, A.J.; Mandl, K.D. Relative Impact of Influenza and Respiratory Syncytial Virus in Young Children. *Pediatrics* **2009**, *124*, e1072–e1080.
34. Johnson, A.J.; Moore, Z.S.; Edelson, P.J.; Kinnane, L.; Davies, M.; Shay, D.K.; Balish, A.; McCarron, M.; Blanton, L.; Finelli, L.; et al. Household responses to school closure resulting from outbreak ofinfluenza B, North Carolina. *Emerg. Infect. Dis.* **2008**, *14*, 1024–1030.
35. Li, S.; Leader, S. Economic burden and absenteeism from influenza-like illness in healthy households with children (5–17 years) in the US. *Respir. Med.* **2007**, *101*, 1244–1250.
36. Ploin, D.; Gillet, Y.; Morfin, F.; Fouilhoux, A.; Billaud, G.; Liberas, S.; Denis, A.; Thouvenot, D.; Fritzell, B.; Lina, B.; et al. Influenza burden in febrile infants and young children in a pediatricemergency department. *Pediatr. Infect. Dis. J.* **2007**, *26*, 142–147.
